# HPV-mediated nuclear export of HP1γ drives cervical tumorigenesis by downregulation of p53

**DOI:** 10.1038/s41418-020-0520-5

**Published:** 2020-03-23

**Authors:** Sang Ah Yi, Dong Hoon Lee, Go Woon Kim, Hyun-Wook Ryu, Jong Woo Park, Jaecheol Lee, Jihoon Han, Jee Hun Park, Hwamok Oh, Jieun Lee, Junjeong Choi, Hyun-Soo Kim, Hyeok Gu Kang, Da-Hyun Kim, Kyung-Hee Chun, Jueng Soo You, Jeung-Whan Han, So Hee Kwon

**Affiliations:** 1 Epigenome Dynamics Control Research Center, School of Pharmacy, Sungkyunkwan University, Suwon, 16419 Republic of Korea; 2 College of Pharmacy, Yonsei Institute of Pharmaceutical Sciences, Yonsei University, Incheon, 21983 Republic of Korea; 3 Department of Pathology, Severance Hospital, Yonsei University College of Medicine, Seoul, 03722 Republic of Korea; 4 Department of Biochemistry and Molecular Biology, Yonsei University College of Medicine, Seoul, 03722 Republic of Korea; 5 Brain Korea 21 PLUS Project for Medical Science, Yonsei University College of Medicine, Seoul, 03722 Republic of Korea; 6 Department of Biochemistry, School of Medicine, Konkuk University, Seoul, 05029 Republic of Korea

**Keywords:** Epigenetics, Tumour-suppressor proteins

## Abstract

E6 oncoprotein derived from high-risk human papillomavirus (HPV) drives the development of cervical cancer through p53 degradation. Because cervical cancer therapies to inactivate HPV or E6 protein are not available, alternative strategies are required. Here, we show that HPV-mediated nuclear export of human heterochromatin protein 1γ (HP1γ) reduces the stability of p53 through UBE2L3-mediated p53 polyubiquitination during cervical cancer progression. In general, HP1 plays a key role in heterochromatin formation and transcription in the nucleus. However, our immunostaining data showed that the majority of HP1γ is localized in the cytoplasm in HPV-mediated cervical cancer. We found that HPV E6 protein drives unusual nuclear export of HP1γ through the interaction between the NES sequence of HP1γ and exportin-1. The mutation of the NES sequence in HP1γ led to nuclear retention of HP1γ and reduced cervical cancer cell growth and tumor generation. We further discovered that HP1γ directly suppresses the expression of *UBE2L3* which drives E6-mediated proteasomal degradation of p53 in cervical cancer. Downregulation of *UBE2L3* by overexpression of HP1γ suppressed UBE2L3-dependent p53 degradation-promoting apoptosis of cervical cancer cells. Our findings propose a useful strategy to overcome p53 degradation in cervical cancer through the blockage of nuclear export of HP1γ.

## Introduction

Cervical carcinogenesis is induced by persistent high-risk human papillomavirus (HPV) infection, which is different from other cancers or malignant diseases [1]. Among the fifteen carcinogenic HPV subtypes, high-risk HPV subtypes, including HPV16 and HPV18, are associated with ~70% of the cervical cancers [2, 3]. Although the incidence and mortality rates of patients with cervical cancer are decreasing due to effective diagnosis and vaccines, these methods do not cure patients who already have cervical cancer [4]. Moreover, currently existing therapeutic options, such as surgery, radiotherapy, and chemotherapy, are limited in patients with advanced or recurrent cervical cancers [5, 6]. Thus, a significant number of studies are struggling to investigate targeted therapeutics using pathogenic mechanisms.

Oncogenic transformations by high-risk HPVs are mediated by the viral E6 and E7 oncogenes, which are regularly co-expressed [7]. The E7 oncoprotein dysregulates the cell cycle by inactivating the retinoblastoma tumor suppressor gene [8]. The E6 protein blocks p53-mediated growth arrest and apoptosis by inducing p53 degradation [9], which is dependent on E3 ubiquitin ligase, E6AP, and E2 ubiquitin-conjugating enzyme UBE2L3 [10–12]. Thus, the restoration of the p53 function by blocking E6 and E6AP-mediated degradation of p53 could be an attractive strategy for treating cervical cancers. However, to date, there have been no agents that can block the E6-specific degradation of p53 in HPV-driven cervical cancers.

Heterochromatin protein 1 (HP1), a histone code reader, specifically recognizes and binds to methylated histone H3 lysine 9 (H3K9me) [13, 14]. The mammalian HP1 family, which comprises three isoforms (HP1α, β, and γ), plays essential roles in various cellular processes including the heterochromatin organization, centromere stability, telomere stability, DNA repair, and cellular senescence [15–18]. HP1 possesses three characteristic structural domains: an amino-terminal chromodomain (CD), a flexible hinge region, and a carboxyl-terminal chromoshadow domain (CSD) [19]. The HP1 CD binds H3K9me [13, 20, 21], whereas the CSD can homo- and heterodimerize and bind proteins containing the consensus sequence PXVXL [19, 22]. HP1 isoforms exhibit different subnuclear localization in interphase: HP1α and HP1β are centromeric whereas HP1γ is in both euchromatic and heterochromatic regions [15, 23], implying that HP1γ has more diverse functions not yet discovered. Several studies have shown that HP1 contributes to the progression of several cancers [24–26], while there are also evidences demonstrating that expression of HP1 isoforms is decreased in diverse tumor tissues [17]. Adding to these controversial findings, an in-depth understanding of the role of HP1 in cancer progression would provide an interesting target for therapy.

Here, we found that abnormal nuclear export of HP1γ, which is mediated by exportin-1, is responsible for E6-dependent p53 degradation. Inhibition of exportin-1 or expression of nuclear export signal (NES)-depleted HP1γ blocked the nuclear export of HP1γ and subsequently restored p53 signaling reducing the tumorigenic potential of cervical cancer cells. Genome-wide analysis identified UBE2L3, which plays a role in p53 degradation, as a target gene of HP1γ. Overexpression of HP1γ suppressed UBE2L3 and restored p53 stability, further inducing apoptosis of cervical cancer cells. These observations prompted us to take a closer look at the role of HP1γ in the cervical cancer and provide a pathogenic rationale to treat cervical cancer.

## Materials and methods

### DNA constructs and antibodies

The DNA constructs used in this study were pcDNA-EGFP-HP1β, pcDNA-EGFP-HP1γ, pcDNA-EGFP-HP1γ V32A, pcDNA-EGFP-HP1γ I165E, pcDNA-EGFP-HP1γ W174A, pcDNA-EGFP-HP1γ 150A, 152A, and pNCMV 16E6no*. The mutant constructs for HP1γ were generated using site-directed mutagenesis (pcDNA-EGFP-HP1γ V32A, pcDNA-EGFP-HP1γ I165E, pcDNA-EGPF-HP1γ W174A, and pcDNA-EGFP-HP1γ 150A, 152A). Anti-HP1α (Epitomics, 5346-1), anti-HP1β (Millipore, MAB3448), anti-HP1γ (Millipore, 05-690), anti-p53 (Santa Cruz Biotechnology, SC-126), anti-UBE2L3 (abcam, ab108936), anti-ubiquitin (Santa Cruz Biotechnology, SC-9133), anti-Exportin-1 (Santa Cruz Biotechnology, SC-74454), anti-Bax (Santa Cruz Biotechnology, SC-20067), anti-Noxa (Santa Cruz Biotechnology, SC-56169), anti-Puma (Santa Cruz Biotechnology, SC-28226), anti-Caspase 3 (abcam, ab13585-100), anti-Flag (Sigma, F-3165), anti-GFP (Santa Cruz Biotechnology, SC-9996), anti-α-tubulin (Santa Cruz Biotechnology, SC-32293), anti-actin (Millipore, mab1501), and anti-Lamin A/C (Cell Signaling Technology, #2032) antibodies were used in this study.

### Cell lines and treatment

HeLa, SiHa, CaSki, and U2OS cells were purchased from the American Type Culture Collection (ATCC). The cells were cultured according to the instructions from ATCC and were maintained under a fully humidified atmosphere of 95% air and 5% CO_2_ at 37 °C. To inhibit exportin-1, cells were treated with 50 nM of leptomycin B (LMB) purchased from Sigma-Aldrich for 4 h or 100 nM of KPT-330 purchased from Selleckchem for 24 h.

### Doxycycline-inducible HP1γ expression

The Tet-on 3G doxycycline-inducible expression system (Takara Bio USA, #631354) was used to promote the expression of *HP1γ* inserted under the TRE3G promoter in SiHa cells according to the manufacturer’s protocol. After cloning HP1γ wild-type (WT) or AA mutant into the pLVX-TRE3G-IRES vector, we gathered lentiviral supernatants from 293T cells expressing both regulator vector (pLVX-EF1a-Tet3G) and response vector (pLVX-TRE3G-IRES-HP1γ WT or pLVX-TRE3G-IRES-HP1γ AA). Then, SiHa cells were coinfected with the two viruses following G418 selection (500 μg/ml). After incubation with or without doxycycline (1 μg/ml) for additional 48 h, the cells were harvested for analyses.

### Knockdown of gene expression

Cells were transfected with siRNA targeting HP1α, HP1β, HP1γ, UBE2L3, or HPV16 E6 using Lipofectamine 2000 reagent (Life Technologies) according to the manufacturer’s protocol. The sequences of siRNAs are as follow:

HP1α sense, 5′-GUUCCAGUCCUCUCUCAAAGC-3′

HP1α antisense, 5′-GCUUUGAGAGAGGACUGGAAC-3′;

HP1β sense, 5′-GACUCCAGUGGAGAGCUCAUG-3′

HP1β antisense, 5′-CAUGAGCUCUCCACUGGAGUC-3′;

HP1γ sense, 5′-AUUCUUCAGGCUCUGCCUC-3′

HP1γ antisense, 5′-GAGGCAGAGCCUGAAGAAU-3′;

UBE2L3 sense, 5′-UUUCUUUGUAAACUCUUCA-3′

UBE2L3 antisense, 5′-UGAAGAGUUUACAAAGAAA-3′;

HPV16 E6 sense, 5′-CCACAGUUAUGCACAGAGC-3′

HPV16 E6 antisense, 5′-GCUCUGUGCAUAACUGUGG-3′.

### Immunohistochemistry

Formalin-fixed and paraffin-embedded tissue sections were acquired from normal people and HPV-positive precancer, endocervical adenocarcinoma, and invasive squamous cell carcinoma patients. The 4-μm sections were deparaffinized in xylene and rehydrated through a graded alcohol series to distilled water. The antigen retrieval was performed by microwave irradiation, and the primary antibody against HP1γ (05-690, Millipore, diluted at 1:200) was then applied. The specific binding was detected with biotinylated anti-mouse immunoglobulin, followed by peroxidase-labeled streptavidin with 3,3′-diaminobenzidine chromogen as substrate. Slides were counterstained with Harris hematoxylin. All protocols and procedures with human cervical tissues were approved by the Yonsei University Institutional Review Board (4-2017-0898).

### Immunoblotting and immunoprecipitation

For immunoblotting, each sample was subjected to SDS-polyacrylamide gel electrophoresis. Proteins were transferred to polyvinylidene difluoride membranes using the semi-dry transfer (Bio-Rad). The membranes were incubated overnight with the indicated primary antibodies, followed by incubation with horseradish peroxidase-conjugated secondary antibodies for 1 h (Abcam). The signals were detected using chemiluminescence reagents (Intron). For immunoprecipitation, the cells were lysed with IP lysis buffer (HEPES 40 mM (pH 7.4) containing 120 mM NaCl, 1 mM EDTA, 50 mM NaF, 1.5 mM Na_3_VO_4_, 10 mM β-glycerophosphate, 0.3% CHAPS, and protease inhibitors). The lysates were centrifuged for 20 min at 13,000 rpm at 4 °C. The specific antibodies were incubated with the supernatants overnight at 4 °C, followed by incubation with anti-rabbit Ig-IP beads (Trueblot) for 1 h at 4 °C. The beads were spun-down for 1 min at 2000 rpm and washed three times with IP wash buffer (IP lysis buffer without CHAPS). The proteins were eluted by boiling for 5 min in Laemmli buffer (Bio-Rad) and subjected to immunoblotting.

### Nuclear fractionation

For the fractionation of cytoplasmic and nuclear extracts, cells were suspended in buffer A (10 mM HEPES containing 1.5 mM MgCl_2_, 10 mM KCl, 1 mM EDTA, 1 mM DTT, 0.5 μg/ml leupeptin, 1 mM PMSF, 1 μM pepstatin A, and 0.05% NP-40), and cytoplasmic extracts were separated by centrifugation at 4 °C at 3000 rpm for 10 min. The remained pellet was resuspended in Buffer B (20 mM HEPES containing 1.5 mM MgCl_2_, 420 mM KCl, 25% glycerol, 0.2 mM EDTA, 1 mM DTT, 0.5 μg/ml leupeptin, 1 mM PMSF, and 1 μM pepstatin A) and incubated on ice for 30 min. Nuclear extracts were separated by centrifugation at 4 °C at 13,000 rpm for 20 min.

### Clonogenic assay

For the stable cells, SiHa cells expressing doxycycline-inducible HP1γ WT or AA were seeded in 1 × 10^3^ cells per well of a six-well plate and cultured with doxycycline (0.5 μg/ml) for 10–20 days. For transiently expressing cells, HeLa or Siha cells were seeded in 2 × 10^3^ cells per well of a six-well plate. Five days after, the cells were transfected with siRNA or DNA plasmids and the cells were grown 3–5 days more. Cells were fixed with glutaraldehyde (6%) and stained with 0.5% crystal violet. The images of stained colonies were visualized using a digital scanner.

### Tumorigenesis with the xenograft mouse model

All animal experiments were approved by the Institutional Review Board of the Yonsei University College of Medicine and were performed in specific pathogen-free facilities according to the university’s guidelines for the Care and Use of Laboratory Animals (2018–0155). Female Balb/c nude mice (5–7 weeks old) were inoculated subcutaneously with 3 × 10^6^ stable SiHa cells expressing pLVX-EF1a-Tet3G (control), pLVX-TRE3G-IRES-HP1γ WT, or pLVX-TRE3G-IRES-HP1γ AA into each flank under 100 µL of saline/zoletil/rompun (7:1:1) anesthesia. Mice were randomized into groups (*n* = 8 per group), and treatment was started 14 days after tumor implantation. Doxycycline (Duchefa, Netherlands) was administered to tumor-bearing animals orally, two times a week for 3 weeks at a dose of 50 mg/kg. From palpable tumor formation until termination, tumor sizes were measured every 3–4 days using calipers, and tumor volume were calculated with the following formula: length × width^2^ × 0.5236. Mice were sacrificed in a 7.5% CO_2_ chamber, and tumors were harvested for immunohistochemistry and other analyses.

### Microarray

RNA labeling and hybridization were performed by using the Agilent One-Color Microarray-Based Gene Expression Analysis protocol (Agilent Technology, V 6.5, 2010). Briefly, 200 ng of total RNA from each sample was linearly amplified and labeled with Cy3-dCTP. The labeled cRNAs were purified by the RNAeasy Mini Kit (Qiagen). The concentration and specific activity of the labeled cRNAs (pmol Cy3/μg cRNA) were measured by NanoDrop ND-1000 (NanoDrop). A total of 600 ng of each labeled cRNA was fragmented by adding 5 μl 10× blocking agent and 1 μl of 25× fragmentation buffer, and then heated at 60 °C for 30 min. Finally, 25 μl of 2× GE hybridization buffer was added to dilute the labeled cRNA. Fifty microliters of hybridization solution was dispensed into the gasket slide and assembled to the SurePrint G3 Human Microarrays, 8 × 60 K (Agilent Technologies, Inc). The slides were incubated for 17 h at 65 °C in an Agilent hybridization oven and then washed at room temperature by using the Agilent One-Color Microarray-Based Gene Expression Analysis protocol (Agilent Technology, V 6.5, 2010). The hybridized array was immediately scanned with an Agilent Microarray Scanner (Agilent Technologies, Inc.). The cDNA microarray experiments (one-channel method) were repeated twice and the average of two gene expression values for each gene was used for further analysis. Statistical significance of the expression data was determined using LPE (Local Pooled Error) test and fold change in which the null hypothesis was that no difference exists among 2 groups. For statistical analysis, the LPE test was used for multiple group comparisons across all samples in the experiment by MeV (MultiExperiment Viewer) Software. A *p* value of <0.05 was considered statistically significant and termed differentially expressed genes.

### Quantitative real-time PCR (qPCR)

Total RNA was extracted using Easy-Blue reagent (iNtRON, Korea). Then 1 μg of total RNA was reverse transcribed into cDNA using a Reverse Transcription kit (Promega, USA). Quantitative real-time PCR was performed using KAPATM SYBR FAST qPCR (KAPABIOSYSTEMS) with a CFX96TM or Chromo4TM real-time PCR detector (Bio-Rad). Relative levels of mRNA were normalized to the values of *GAPDH* mRNA for each reaction. The qPCR primer sequences used are as follow:

*GAPDH* forward, 5′-GAGTCAACGGATTTGGTCGT-3′;

*GAPDH* reverse, 5′-TTGATTTTGGAGGGATCTCG-3′;

*TP53* forward, 5′-GAGGGATGTTTGGGAGATGTAAGAAATG-3′;

*TP53* reverse, 5′-TTCACAGATATGGGCCTTGAAGTTAGAGAA-3′;

*UBE2L3* forward, 5′-TTGACCCTT TGTAGGATTGGAATT-3′;

*UBE2L3* reverse, 5′-CGACCCCAGACTGGTGCTT-3′;

*Bax* forward, 5′-TCTACTTTGCCAGCAAACTGG-3′;

*Bax* reverse, 5′-TGTCCAGCCCATGATG GTTCT-3′;

*Noxa* forward, 5′-AGAGCTGGAAGTCGAGTGT-3′;

*Noxa* reverse, 5′-GCACCT TCACATTCCTCTC-3′;

*Puma* forward, 5′-GACCTCAACGCACAGTA-3′;

*Puma* reverse, 5′-CTAATTGGGCTCCATCT-3′;

*TP53AIP1* forward, 5′-TCTTCCTCTGAGGCGAGCT-3′;

*TP53AIP1* reverse, 5′-AGGTGTGTGTGTCTGAGCCC-3′;

*14-3-3σ* forward, 5′-TTTCCTCT CCAGACTGACAAACTGTT-3′;

*14-3-3σ* reverse, 5′-TAGAACTGAGCTGCAGCTGTAAA -3′;

*Gadd45α* forward, 5′-TGCGAGAACGACATCAACAT-3′;

*Gadd45α* reverse, 5′-TCCCG GCAAAAACAAATAAG-3′;

*p21* forward, 5′-CACCGAGACACCACTGGAGG-3′;

*p21* reverse, 5′-GAGAAGATCAGCCGGCGTTT-3′;

*Tp53inp1* forward, 5′-TGTTGCAGCTCTT GCTGCTCA-3′;

*Tp53inp1* reverse, 5′-GCTGATGAACAACCCAGCCAT-3′;

*HPV16 E6* forward, 5′-GAGTCAACGGATTTGGTCGT-3′;

*HPV16 E6* reverse, 5′-CATAAATCCCGAAAAGCAAAG-3′.

### Chromatin immunoprecipitation and qPCR (ChIP-qPCR)

Total ChIP was performed as previously described [27]. In brief, a small portion of the cross-linked, sheared chromatin solution was reserved as the input DNA and the remainder was subjected to immunoprecipitation overnight at 4 °C using antibodies. After immunoprecipitation, the recovered chromatin fragments were subjected to qPCR using primer pairs specific for the target gene promoter. The primer sequences used for ChIP-qPCR are as follow:

*Bax* forward, 5′-TAATCCCAGCGCTTTGGAAG-3′;

*Bax* reverse, 5′-TGCAGAGACCTGGATCTAGCA-3′;

*Noxa* forward, 5′-CCTGGCCCCACCCCACCCCAC-3′;

*Noxa* reverse, 5′-TCAGGGCTATTTTACGCTCTC-3′;

*14-3-3σ* forward, 5′-TTTCCTCTCCAGACTGACAAA-3′;

*14-3-3σ* reverse, 5′-TAGAACTGAGCTGCAGCTGTA-3′;

*Gadd45α* forward, 5′-AGCGGAAGAGATCCCTGTGA-3′;

*Gadd45α* reverse, 5′-CGGGAGGCAGGCAGATG-3′.

### Flow cytometry

Apoptosis of cervical cancer cells was assessed using Annexin V-PI double staining as previously described [28]. Cells were trypsinized and stained with 0.5 mg/ml Annexin V in binding buffer (10 mM HEPES free acid, 0.14 M NaCl, and 2.5 mM CaCl_2_) for 15 min. Afterward, PI (5 mg/mL final concentration) was added and incubated for another 15 min, then applied to a flow cytometer for data collection.

### Survival analysis of cervical cancer patients

The normalized level 3 TCGA RNA sequencing data of cervical cancer were retrieved from https://gdac.broadinstitute.org. A total of 61 patients with available clinical information was analyzed for overall survival and disease-free survival. Kaplan–Meier survival curves and log-rank statistics were employed to evaluate time to tumor recurrence and overall survival. Statistical significance was when *p* < 0.05.

### Statistical analysis

Statistical significance was analyzed using Student’s *t* test (two-tailed) and Pearson’s correlation test. Statistical differences were determined based on a *P* value (*p < 0.05, **p < 0.01, and ***p < 0.001).

## Results

### Abnormal nuclear export of HP1γ is induced by high-risk HPV E6 protein

**Fig. 1:**
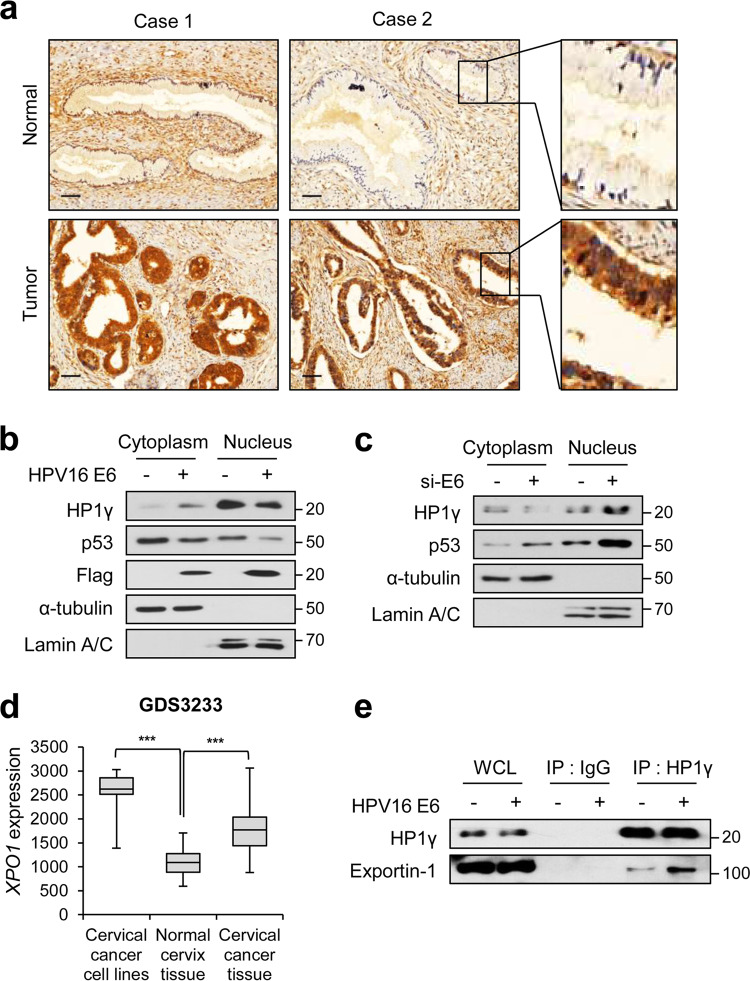
Aberrant nuclear export of HP1γ is induced by high-risk HPV E6 protein. **a** Immunohistochemical staining of HP1γ protein in cervical tissues from normal specimens (upper) and HPV-positive cervical adenocarcinoma patients (lower). **b** Immunoblot analysis of cytoplasmic and nuclear extracts from C33A cells expressing Flag-HPV16 E6. **c** Immunoblot analysis of cytoplasmic and nuclear extracts from SiHa cells expressing siRNA of HPV16 E6. **d** Box-and-Whisker plot of relative *XPO1* expression in cervical cancer cell lines (*n* = 9), normal tissues (*n* = 24), and cervical cancer tissues (*n* = 28) (GDS3233). **e** Immunoblot analysis of HP1γ immunoprecipitates (IP) and whole-cell lysates (WCL) from C33A cells expressing Flag-HPV16 E6.

To gain insight into the putative role of HP1 on cancer progression, we examined the subcellular localization of three HP1 isoforms in diverse cancer samples by analyzing publicly available immunohistochemistry data of The Human Protein Atlas (HPA) project. HPA analysis showed cervical cancer-specific cytoplasmic localization of HP1γ, whereas no distinctive cancer type-specific localization of HP1α and HP1β was observed (Supplementary Fig. S1). Thus, we subjected cervical cancer tissue specimens to immunohistochemical analysis for the staining of HP1γ. Unlike that observed in normal specimens, in which HP1γ was detected mainly in the nuclear region (Fig. 1a, upper), HP1γ was diffused from the nucleus to the cytoplasmic region in HPV-positive adenocarcinoma specimens (Fig. 1a, lower). However, HP1γ in HPV-positive cervical tissues from the precancer stage was detected only in the nucleus, whereas cytoplasmic localization of HP1γ in cervical tissues was observed in both adenocarcinoma and squamous cell carcinoma (Supplementary Fig. S2). TCGA survival analysis showed that the survival of cervical cancer patients was not significantly associated with the expression level of *CBX3* (a gene encoding HP1γ) (Supplementary Fig. S3). These data indicate that subcellular localization of HP1γ, rather than its expression, is closely involved in the progression of cervical cancer.

We next assessed whether the E6 oncoprotein of high-risk HPV is responsible for the nuclear export of HP1γ. Nuclear fractionation analysis after transfection of HPV16 E6 showed an increase in the level of cytoplasmic HP1γ and a reduction in the level of nuclear HP1γ, both in HPV-negative (C33A) and -positive (SiHa) cells (Fig. 1b, Supplementary Fig. S4A), implying immediate nuclear export of HP1γ by high-risk HPV infection. Furthermore, knockdown of E6 protein using siRNA targeting *HPV16 E6* in SiHa cells (Supplementary Fig. S4B) blocked the nuclear export of HP1γ (Fig. 1c). Several studies have reported that E6 protein from HPV forces nuclear export of p53, which leads to degradation of p53 by cytoplasmic proteasomes [29–31]. We also observed that HPV E6 contributed to the reduction of p53 protein levels in both subcellular parts, cytoplasm and nucleus (Fig. 1b, c). While a majority of HP1γ research has focused on its chromatic role, our data provide insights into the cytoplasmic localization of HP1γ, which occurs during HPV infection-mediated cancer progression.

### Exportin-1-mediated nuclear export of HP1γ is responsible for p53 downregulation in cervical cancer

**Fig. 2:**
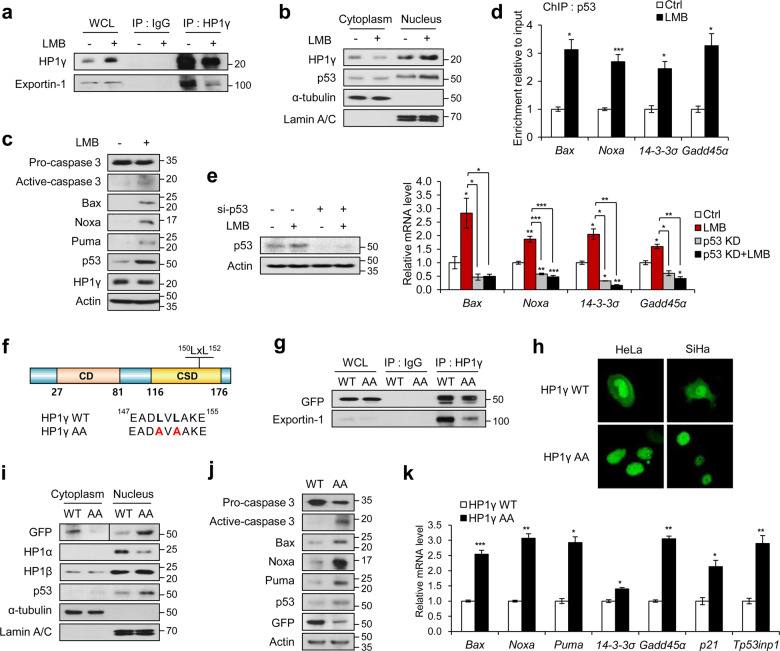
Exportin-1-mediated nuclear export of HP1γ contributes to p53 degradation. **a** Immunoblot analysis of HP1γ immunoprecipitates (IP) and whole-cell lysates (WCL) from HeLa cells treated with or without LMB. **b** Immunoblot analysis of cytoplasmic and nuclear extracts from SiHa cells treated with or without LMB. **c** Immunoblot analysis of SiHa cells treated with or without LMB. **d** SiHa cells were treated with or without LMB, followed by ChIP-qPCR analysis with a p53 antibody in the promoter regions of p53 target genes. **e** Immunoblot analysis and the mRNA levels of p53 target genes in SiHa cells expressing siRNA of p53 in the presence or absence of LMB. **f** Representation of HP1γ protein and partial CD sequence containing nuclear export signal (NES) sequences (L150 and L152) which are substituted with alanine in the mutant vector, HP1γ AA. **g** Immunoblot analysis of HP1γ immunoprecipitates (IP) and whole-cell lysates (WCL) from SiHa cells expressing GFP-HP1γ WT or AA mutant. **h** Immunofluorescence image of GFP-HP1γ WT or AA mutant expressed in HeLa (left) and SiHa (right) cells. **i** Immunoblot analysis of cytoplasmic and nuclear extracts from SiHa cells expressing GFP-HP1γ WT or AA mutant. **j** Immunoblot analysis of SiHa cells expressing GFP-HP1γ WT or AA mutant. **k** The mRNA levels of p53 target genes in SiHa cells expressing GFP-HP1γ WT or AA mutant. Data are presented as the mean ± SEM (*n* = 3). *P < 0.05; **P < 0.01; ***P < 0.001.

Earlier studies have reported that exportin-1 (encoded by *XPO1*) is involved in high-risk HPV-mediated elevation of the nuclear export system [29–32]. Moreover, the analysis of GEO profiles (GDS3233) showed a significant increase in the expression of *XPO1* in cervical cancer cell lines and cervical cancer tissues, compared with normal cervix epithelium tissues (Fig. 1d). In parallel with this finding, the binding of HP1γ to exportin-1 was significantly increased after the overexpression of HPV16 E6 protein (Fig. 1e). This interaction was ablated by treatment with exportin-1 inhibitor, LMB (Fig. 2a), which subsequently induced the nuclear accumulation of HP1γ (Fig. 2b). Nuclear export of p53 was also inhibited by LMB treatment (Fig. 2b), which upregulated the p53 protein level and p53-mediated transcription of its target genes such as *Bax*, *Noxa*, *14-3-3σ*, and *Gadd45α* (Fig. 2c–e). LMB-mediated increase in p53 target gene expression was disturbed by knockdown of p53 (Fig. 2e). Similar effects were produced by KPT-330, another exportin-1 inhibitor (Supplementary Fig. S4C, D). These results suggest that exportin-1 is responsible for the abnormal nuclear export of HP1γ as well as p53.

Given that exportin-1 usually interacts with the leucine-rich site of its target protein, known as a NES sequence [33], we generated an NES mutant of HP1γ containing double alanine mutation (AA) at leucine residues 150 and 152 (Fig. 2f). The interaction between HP1γ AA mutant and exportin-1 was markedly reduced compared with WT HP1γ (Fig. 2g), accompanied by the nuclear accumulation of the HP1γ AA mutant as assessed by immunofluorescence (Fig. 2h) and cell fractionation (Fig. 2i). Intriguingly, expression of the HP1γ AA mutant led to increases in protein levels of p53 and apoptotic markers (Fig. 2j) as well as the transcription of p53 target genes (Fig. 2k), compared with those in WT HP1γ-expressing cells. Thus, the interaction between exportin-1 and the NES of HP1γ transports HP1γ from the nucleus to the cytoplasm, likely involved in the reduction of p53.

### Inhibiting nuclear export of HP1γ ameliorates tumorigenesis of cervical cancer

**Fig. 3:**
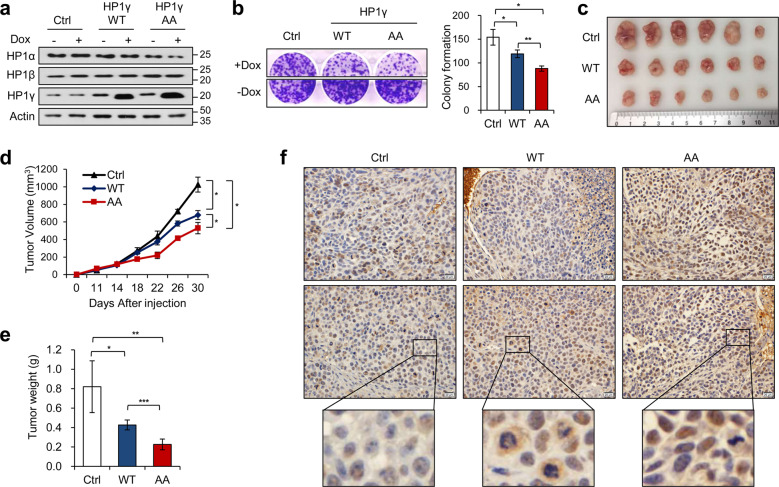
Inhibiting the nuclear export of HP1γ promotes p53 effects in cervical cancer cells. **a** Immunoblot analysis of SiHa cells expressing doxycycline-induced HP1 WT or AA treated with or without doxycycline for 48 h. **b** Clonogenic assay of SiHa cells expressing doxycycline-induced HP1 WT or AA treated with or without doxycycline for 20 days. **c–e** In vivo tumorigenesis assay of SiHa cells was performed. SiHa cells expressing HP1 WT or AA were injected into the flank of nude mice. **f** Immunohistochemical staining of HP1γ protein in tumor tissues derived from mice injected with SiHa cells expressing HP1 WT or AA. Data are presented as the mean ± SD (*n* = 6). *P < 0.05; **P < 0.01; ***P < 0.001.

To investigate whether nuclear export of HP1γ is involved in tumorigenesis of cervical cancer, we generated stable cell lines overexpressing HP1γ using doxycycline-inducible system (Fig. 3a). In line with the data from transiently HP1γ-expressing cells (Fig. 2i), stable expression of HP1γ AA slightly decreased the protein level of HP1α without alterations in the level of HP1β (Fig. 3a). Clonogenic assay data showed that overexpression of WT HP1γ markedly reduced colony formation of SiHa cells, which was further suppressed by long-term expression of HP1γ AA mutant (Fig. 3b). Next, we evaluated the in vivo tumorigenesis of HP1γ-expressing SiHa cells in the xenograft mouse model. The tumorigenic capacity of SiHa cells was impeded by overexpression of HP1γ, particularly in the HP1γ AA mutant group (Fig. 3c). Quantification of tumor volume and tumor weight showed a significant reduction in tumor growth in the HP1γ AA mutant group, and a lesser extent in WT HP1γ group (Fig. 3d, e). We then observed the expression pattern of HP1γ by staining the histological sections of tumor tissues from the xenograft mice. While WT HP1γ was diffused to the cytoplasm in tumor cells (Fig. 3f, middle), the HP1γ AA mutant was predominantly localized at the nucleus (Fig. 3f, right). These data imply the unidentified role of HP1γ in the nucleus to prevent tumor development.

### *UBE2L3* is a candidate target gene of HP1 isoforms in cervical cancer cells

**Fig. 4:**
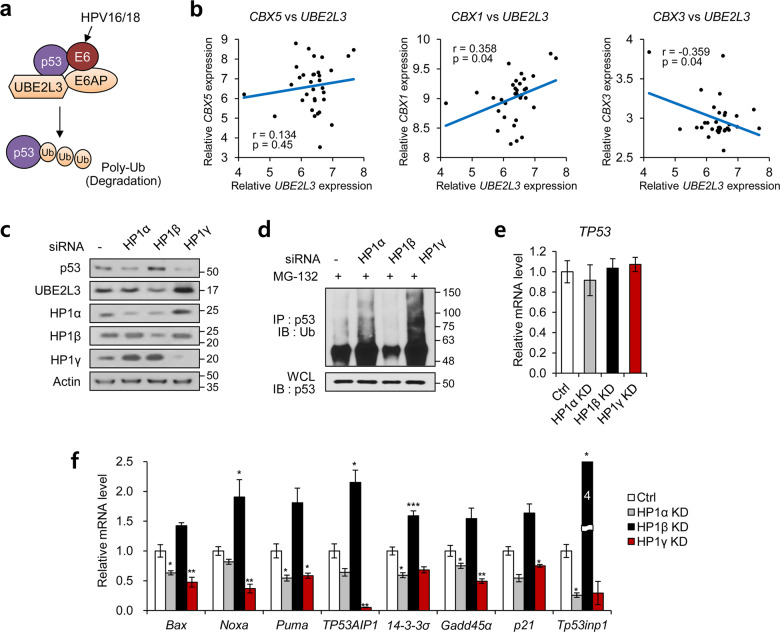
HP1 isoforms regulate UBE2L3-dependent p53 ubiquitination in cervical cancer cells. **a** Model for UBE2L3-mediated p53 degradation upon high-risk HPV infection. **b** Pearson’s correlation analysis in different areas of cervical cancer tumors (GDS2416) (*n* = 33). **c** Immunoblot analysis of HeLa cells expressing siRNA of HP1 isoforms. **d** Immunoblot analysis of p53 immunoprecipitates (IP) and whole-cell lysates (WCL) from HeLa cells expressing siRNA of HP1 isoforms after MG-132 treatment. **e** The mRNA levels of *TP53* in HeLa cells expressing siRNA of HP1 isoforms. **f** The mRNA levels of p53 target genes in HeLa cells expressing siRNA of HP1 isoforms. Data are presented as the mean ± SEM (*n* = 3). *P < 0.05, **P < 0.01, ***P < 0.001.

Based on the altered subcellular localization of HP1γ in cervical cancer, we reasoned that there would be a variety of changes in gene expression, under the control of HP1γ. Thus, we performed a genome-wide microarray analysis of HP1-depleted HeLa cells (Supplementary Fig. S5, Supplementary Tables S1–S7). Among the candidate target genes commonly modulated by the three HP1 isoforms (Supplementary Table S1), *UBE2L3* is known to be involved in proteasomal degradation of p53 during cervical cancer progression, interacting with E6AP to form ubiquitination machinery (Fig. 4a) [34, 35]. Indeed, we observed suppressed *UBE2L3* expression and enhanced p53 signaling upon knockdown of HPV E6 (Supplementary Fig. S6). Depletion of UBE2L3 elevated p53 signaling, accordingly reducing cell growth rate and colony forming ability of cervical cancer cells (Supplementary Fig. S7). Analysis of GEO profiles (GDS2416) in 33 different biopsies from 16 cervical cancer patients showed a negative correlation between the expression of *UBE2L3* and *CBX3* (a gene encoding HP1γ), whereas the expression of *UBE2L3* and *CBX1* (a gene encoding HP1β) showed a positive correlation (Fig. 4b). There was no statistically significant correlation between *UBE2L3* and *CBX5* (a gene encoding HP1α) levels (Fig. 4b). None of the three HP1 isoforms showed a correlation with the expression of *UBE3A*, a gene coding E6AP (Supplementary Fig. S8). The ChIP-qPCR analysis showed that the three HP1 isoforms were differentially recruited to the promoter of *UBE2L3* as well as other candidate target genes (Supplementary Fig. S5D, E), indicating that *UBE2L3* is a direct target of the HP1 isoforms. In addition, the protein level of UBE2L3 was elevated in HP1γ knockdown cells, whereas the depletion of HP1β led to an unexpected reduction of UBE2L3 protein (Fig. 4c). Consequently, the protein level of p53 exhibited an inverse correlation with the UBE2L3 level (Fig. 4c), which was resulted from alterations in p53 protein stability (Fig. 4d). None of the HP1γ or HP1β knockdown affected the *TP53* mRNA level (Fig. 4e), demonstrating that the change in p53 protein level upon HP1 depletion did not originate from the regulation of its transcription. Moreover, HP1γ depletion downregulated expression of p53 target genes, whereas HP1β depletion produced the opposite effect (Fig. 4f). The effects of HP1α depletion were similar to but weaker than those of HP1γ depletion. These results indicate that HP1 isoforms differentially regulate the transcription of the *UBE2L3* gene.

### HP1γ is a major regulator of UBE2L3-dependent p53 ubiquitination

**Fig. 5:**
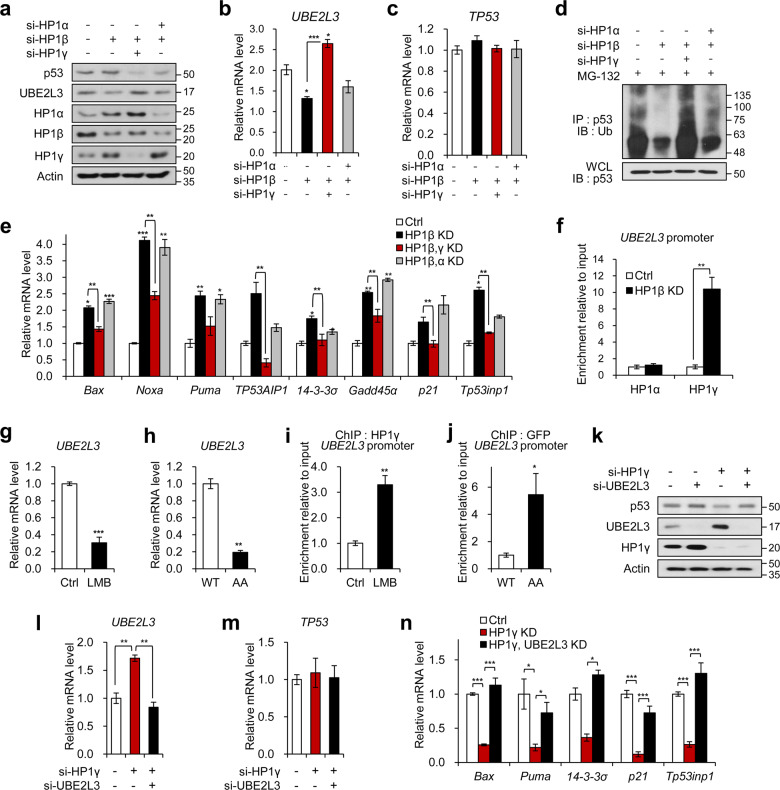
HP1γ negatively regulates p53 ubiquitination by suppressing *UBE2L3* expression. **a** Immunoblot analysis of HeLa cells co-expressing siRNA of HP1 isoforms. **b** The mRNA levels of *UBE2L3* in HeLa cells co-expressing siRNA of HP1 isoforms. **c** The mRNA levels of *TP53* in HeLa cells co-expressing siRNA of HP1 isoforms. **d** Immunoblot analysis of p53 immunoprecipitates (IP) and whole-cell lysates (WCL) from HeLa cells co-expressing siRNA of HP1 isoforms after MG-132 treatment. **e** The mRNA levels of p53 target genes in HeLa cells co-expressing siRNA of HP1 isoforms. **f** HeLa cells were transfected with siRNA of HP1β, followed by ChIP-qPCR analysis for HP1α and HP1γ antibodies in the promoter region of the *UBE2L3* gene. **g** SiHa cells were treated with or without LMB, followed by ChIP-qPCR analysis with an HP1γ antibody in the promoter region of the *UBE2L3* gene. **h** The mRNA levels of *UBE2L3* in SiHa cells treated with or without LMB. **i** SiHa cells were transfected with GFP-HP1γ WT or AA mutant vectors, followed by ChIP-qPCR analysis with a GFP antibody in the promoter region of *UBE2L3* gene. **j** The mRNA levels of *UBE2L3* in SiHa cells expressing GFP-HP1γ WT or AA mutant. **k** Immunoblot analysis of HeLa cells co-expressing siRNA of HP1γ and UBE2L3. **l** The mRNA levels of *UBE2L3* in HeLa cells co-expressing siRNA of HP1γ and UBE2L3. **m** The mRNA levels of *TP53* in HeLa cells co-expressing siRNA of HP1γ and UBE2L3. **n** The mRNA levels of p53 target genes in HeLa cells co-expressing siRNA of HP1γ and UBE2L3. Data are presented as the mean ± SEM (*n* = 3). *P < 0.05, **P < 0.01, ***P < 0.001.

As the depletion of each HP1 isoform led to a reciprocal increase in the expression of other HP1 isoforms (Fig. 4c), we hypothesized that the reduction in the UBE2L3 protein level by HP1β depletion would be produced through a compensatory response by HP1α or HP1γ. To test this, we examined the effect of co-silencing of HP1β with another HP1 isoform, HP1α or HP1γ, on the UBE2L3 and p53 protein levels. A reduction in UBE2L3 expression by HP1β depletion was recovered by co-knockdown of HP1γ, but not HP1α (Fig. 5a, b), further leading to the reduction in p53 protein level that was elevated by depletion of HP1β alone (Fig. 5a). This effect was mediated by the alteration of ubiquitination, but not the mRNA level of *TP53* (Fig. 5c, d). The elevation of the expressions of p53 target genes by HP1β knockdown was also antagonized by HP1γ co-knockdown (Fig. 5e). Critically, HP1β depletion significantly increased the recruitment of HP1γ, but not HP1α, to the promoter of *UBE2L3* (Fig. 5f), indicating that the upregulation of the p53 protein by HP1β depletion was due to the suppression of *UBE2L3* expression through compensation by HP1γ. Indeed, inhibiting nuclear export of HP1γ using LMB or AA mutant successfully suppressed *UBE2L3* expression (Fig. 5g, h) by recruitment of HP1γ to the *UBE2L3* promoter (Fig. 5i, j). In contrast, knockdown of HP1α did not lead to compensatory recruitment of HP1γ to the promoter of *UBE2L3* (Supplementary Fig. S9A).

To explore whether the upregulation of p53 by HP1γ is dependent on *UBE2L3* suppression, we observed the effects of HP1γ knockdown in UBE2L3-depleted HeLa cells (Fig. 5k, l). The reduction of p53 protein level by HP1γ knockdown was reversed by the co-silencing of UBE2L3, without changing mRNA level of *TP53* (Fig. 5k, m), indicating that the suppression of *UBE2L3* expression is required for HP1γ-mediated upregulation of p53. Consistently, decreased expressions of p53 target genes upon HP1γ knockdown were recovered by the co-knockdown of UBE2L3 (Fig. 5n). However, the co-knockdown of HP1α and UBE2L3 partially recovered HP1α knockdown-mediated inhibition of p53 signaling (Supplementary Fig. S9B, C).

To investigate whether HP1s-mediated UBE2L3 alteration is dependent on HPV E6, we tested the effects of ectopic expression of HPV16 E6 in HPV-negative cancer cells (U2OS). Although E6 expression in U2OS cells lowered the level of HP1α and HP1β while upregulating HP1γ expression (Supplementary Fig. S10A, B), enrichment of HP1γ on UBE2L3 promoter significantly decreased (Supplementary Fig. S10C). Taken together, these data demonstrate that HP1γ mainly suppresses *UBE2L3* expression to elevate p53 signaling, which is dependent on HPV E6.

### Overexpression of HP1γ promotes p53 effects by directly suppressing *UBE2L3* expression in an HPV-positive manner

**Fig. 6:**
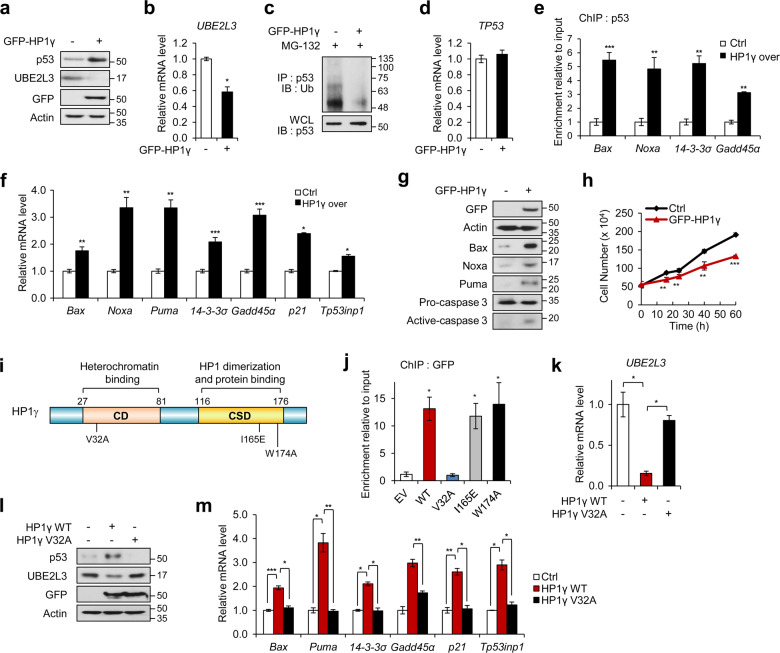
Overexpression of HP1γ promotes p53 signaling by directly suppressing *UBE2L3* expression. **a** Immunoblot analysis of HeLa cells expressing GFP-HP1γ. **b** The mRNA levels of *UBE2L3* in HeLa cells expressing GFP-HP1γ. **c** Immunoblot analysis of p53 immunoprecipitates (IP) and whole-cell lysates (WCL) from HeLa cells expressing GFP-HP1γ after MG-132 treatment. **d** The mRNA levels of *TP53* in HeLa cells expressing GFP-HP1γ. **e** ChIP-qPCR analysis with a p53 antibody in the promoter regions of p53 target genes in HeLa cells expressing GFP-HP1γ. **f** The mRNA levels of p53 target genes in HeLa cells expressing GFP-HP1γ. **g** Immunoblot analysis of HeLa cells expressing GFP-HP1γ. **h** The growth rate of HeLa expressing GFP-HP1γ. **i** Schematic representation of HP1γ protein and partial CD sequence containing V32. **j** HeLa cells were transfected with GFP-HP1γ WT or mutant (V32A, I165E, or W174A) vectors, followed by ChIP-qPCR analysis with a GFP antibody in the promoter regions of the *UBE2L3* gene. **k** The mRNA levels of *UBE2L3* in HeLa cells expressing GFP-HP1γ wild-type (WT) or V32A mutant. **l** Immunoblot analysis of HeLa cells expressing GFP-HP1γ WT or V32A mutant. **m** The mRNA levels of p53 target genes in HeLa cells expressing GFP-HP1γ WT or V32A mutant. Data are presented as the mean ± SEM (*n* = 3). *P < 0.05; **P < 0.01; ***P < 0.001.

Next, we assessed whether HP1γ overexpression prevents UBE2L3-mediated p53 degradation. Overexpression of HP1γ in HeLa cells blocked the expression of UBE2L3 (Fig. 6a, b) and ubiquitination of p53 (Fig. 6c), resulting in an increase in p53 protein level (Fig. 6a) but not *TP53* mRNA (Fig. 6d). Moreover, p53 was significantly recruited to the promoters of its target genes (Fig. 6e), leading to the upregulation of their expression (Fig. 6f). In addition, ectopic expression of HP1γ induced the expression of apoptotic markers (Fig. 6g) and reduced the growth rate of HeLa cells (Fig. 6h). The positive effects of HP1γ overexpression on p53 function were further confirmed by similar observations in other HPV-positive cervical cancer cell lines, SiHa, and CaSki (Supplementary Fig. S11). However, in HPV-negative cell lines (C33A HPV-negative cervical cancer cells and U2OS osteosarcoma cells), HP1γ overexpression had no effect on the protein levels of UBE2L3 and p53 (Supplementary Fig. S12A) as well as the expression of p53 target genes (Supplementary Fig. S12B). Interestingly, HP1γ overexpression in C33A and U2OS cells ectopically expressing HPV16 E6 produced similar effects observed in HPV-positive cervical cancer cells (Supplementary Fig. S12C, D). These data indicate that the role of ectopically expressed HP1γ in p53 elevation is specific for high-risk HPV.

Next, we checked whether overexpression of HP1β affects the regulatory function of HP1γ in suppression of UBE2L3. Overexpression of HP1β increased the level of p53 and its target genes (Supplementary Fig. S13A, B), but UBE2L3 level was not altered by HP1β overexpression (Supplementary Fig. S13A, C). There was a slight synergy effect by co-overexpressing HP1β and HP1γ on induction of p53 signaling and inhibition of colony formation of cervical cancer cells (Supplementary Fig. S13A, B, D), while UBE2L3 suppression by HP1γ was not affected by co-expression of HP1β (Supplementary Fig. S13C). These data suggest that HP1β induces p53 function through an independent mechanism of UBE2L3.

Since we observed that HP1γ recruited to the promoter region of the *UBE2L3* gene (Fig. 5F), we tested whether chromatin binding of HP1γ is critical for the suppression of *UBE2L3* expression. We generated an HP1γ CD mutant possessing a single-site mutation from valine 32 (V32) to alanine (Fig. 6i), which leads to a failure in chromatin binding [36]. As expected, HP1γ V32A was not recruited to the promoter region of *UBE2L3*, whereas ectopically expressed WT HP1γ were enriched on the promoter region (Fig. 6j). Unlike WT HP1γ, HP1γ V32A failed to suppress *UBE2L3* expression (Fig. 6k, l), elevate the p53 protein level (Fig. 6l), and promote transcription of p53 target genes (Fig. 6m). These data suggest that the binding of HP1γ to the promoter region of the *UBE2L3* gene is required for suppression of the gene and subsequent induction of p53 signaling.

To further examine the functional involvement of the CSD in this response, we also produced two CSD mutants of HP1γ (Fig. 6i). The HP1γ I165E mutant disrupts homodimerization, while the HP1γ W174A mutant fails to interact with other proteins containing the PXVXL motif [19, 37]. Despite substantial chromatin binding of both HP1γ CSD mutants (Fig. 6j), neither HP1γ I165E nor HP1γ W174A suppressed UBE2L3 expression, elevated the p53 protein level (Supplementary Fig. S14A–C), and reduced polyubiquitination of p53 (Supplementary Fig. S14D). Consistently, both mutants were unable to induce apoptotic markers (Supplementary Fig. S14A), expression of p53 target genes (Supplementary Fig. S14E), and the growth arrest of cervical cancer cells (Supplementary Fig. S14F). In addition, flow cytometric analysis showed an increase in the proportion of apoptotic cells among cells expressing WT but not mutants HP1γ (Supplementary Fig. S14G–I). Taken together, these results indicate that all the domains of HP1γ are required for the suppression of *UBE2L3* expression and subsequent upregulation of p53.

## Discussion

The role of HP1γ in euchromatic gene regulation as well as in heterochromatic formation has been suggested, because of its unique subnuclear localization [15, 23]. Based on the hypothesis that the three HP1 isoforms differentially regulate gene expression, we explored the effects of HP1 depletion on gene regulation throughout the human genome. Within the commonly regulated genes, we identified an interesting target gene, *UBE2L3*, which contributes to HPV-mediated proteasomal degradation of p53. Despite the overlapping gene regulation in the microarray screening, our data demonstrate that HP1γ is a key suppressor of *UBE2L3* expression (Fig. 4). Although HP1α and HP1β also increased p53 level, they were not closely related to HPV E6-mediated UBE2L3 induction or HP1γ-mediated UBE2L3 suppression. The regulation of p53 by HP1α was partially dependent on UBE2L3 (Supplementary Fig. S9), while HP1β seemed to regulate p53 through a separate mechanism from UBE2L3 (Supplementary Fig. S10). These results suggest that the involvement of each HP1 isoform in UBE2L3-p53 axis is different. A previous study has revealed that the length of the hinge region connecting CD and CSD determines the distinct binding properties of HP1 isoforms [38]. Thus, a relatively short hinge region of HP1γ compared with that of HP1α and HP1β appears to be contributing to the HP1γ’s specific action on *UBE2L3* gene regulation.

Many diseases are associated with a dysfunction of ubiquitin signaling, with the E3 ligases being the focus. However, recent evidence demonstrates that mutations or impairments of the E2-conjugating enzymes can lead to severe diseases [39]. Given their relevance to diseases, E2-conjugating enzymes may serve as an important family of therapeutic targets. The functional roles of several E2s in carcinogenesis have been reported [40]. UBE2L3 is one of the most abundant E2s in mammalian cell lines [41] and associated with diverse cancers including hepatocellular carcinoma [42], oral cancer [43], prostate cancer [44], and non-small cell lung cancer [45]. Our findings show that *UBE3L3* is transcriptionally repressed by HP1γ, which is impaired in cervical cancer cells due to the nuclear export of HP1γ. Hence, UBE2L3 might be a potential therapeutic target to treat cancers that are characterized by elevated UBE2L3 expression.

**Fig. 7:**
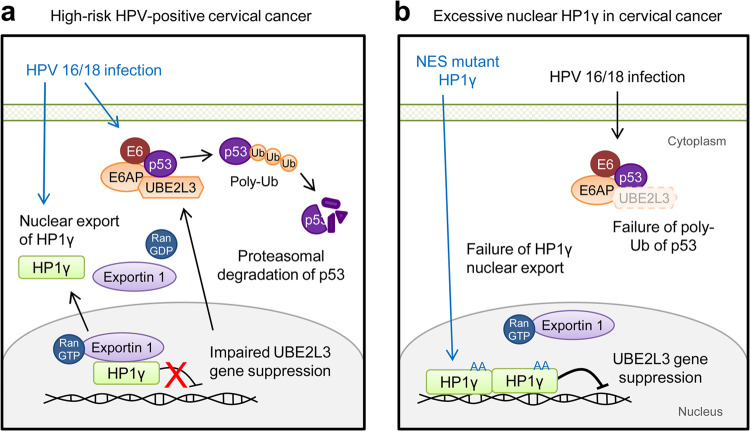
Molecular model describing p53 regulation by HP1γ-dependent UBE2L3 suppression. **a** Upon high-risk HPV infection, the E6 oncoprotein of HPV interacts with p53 and E6AP to induce polyubiquitination and proteasomal degradation of p53. Moreover, HPV infection induces exportin-1-mediated nuclear export of HP1γ, thereby allowing plenty of UBE2L3 to be expressed, which results in p53 degradation in an HPV-specific manner. **b** When the NES mutant HP1γ (HP1γ AA) is overexpressed artificially in cervical cancer cells, exportin-1 fails to transport HP1γ AA to the cytoplasm and HPV-specific degradation of p53 is impaired due to UBE2L3 suppression by nuclear HP1γ.

During the development of cervical cancer, oncogenic HPV E6 plays a central role by disabling the tumor-suppressing function of p53 [11]. In this process, E6AP interacts with UBE2L3, and this complex participates in p53 ubiquitination [10, 12]. Hence, inhibiting the E6-mediated degradation of p53 could be a potent strategy to combat cervical cancer. Recent studies have identified several strategies to inactivate E6 and E7 using RNAi, TALEN, or CRISPR-mediated gene disruption [46–49] or small molecules [50, 51]. However, the previous strategies were limited to in vitro and mouse models and have not yet been tested in human trials [46–51]. In this current study, we present a new approach to protect p53 from E6-mediated degradation by controlling *UBE2L3* expression with HP1γ. HP1γ negatively regulates the ubiquitin-proteasome system (UPS)-dependent p53 degradation through the transcriptional repression of *UBE2L3* while increasing overall p53 protein levels and restoring p53 function (Fig. 7). Intriguingly, these effects of HP1γ were observed only in HPV-positive cervical cancer cells but not HPV-negative cancer cells. Considering that E6AP, rather than MDM2, is a major E3 ubiquitin ligase in HPV-infected cervical cancer cells [52], the effects of HP1γ we observed here was most likely due to suppression of UBE2L3-E6AP-p53 UPS. In line with the previous finding, HPV-positive cervical cancer cells eventually died after overexpression of HP1γ, suggesting that even a partial rescue of p53 is sufficient to induce apoptosis.

Another important issue we found here is the novel role of high-risk HPV E6 in cervical cancer progression through the exportin-1-mediated nuclear export of HP1γ. Although a few papers have proposed the presence of HP1γ in the cytoplasm during maternal to embryonic transition [53] or in myoblasts [54], disease-specific subcellular migration of HP1γ has not been reported yet. In the case of cervical cancer cells, HPV E6 utilizes various Karyopherin proteins for nuclear export and import, after which it can interact with nuclear transcription factors such as p53 [55]. The stability and nuclear export of p53 have been reported to rely on chromosomal regional maintenance-1/exportin-1 activity [29], which is promoted by high-risk HPV E6 [30]. Therefore, exportin-1 is very critical for cancer cell survival and high expression of exportin-1 has been reported in cervical cancer [56]. Interestingly, we found abnormal cytoplasmic localization of HP1γ, which results from increased binding with exportin-1, in HPV-infected cervical cancer cells and tissues. Since HP1γ is observed only within the nucleus in the precancer stage, chronic leakage of HP1γ to the cytoplasm upon persistent infection with high-risk HPV might be an important step in the cancer progression of HPV-infected cells. Notably, inhibition of the nuclear export of HP1γ rescued the p53 level and suppressed tumor growth. These findings suggest that the enhancement of nuclear retention of HP1γ by pharmacological intervention would offer a potential therapeutic strategy to overcome HPV-specific p53 degradation in cervical cancer.

## Supplementary information

Figure S1

Figure S2

Figure S3

Figure S4

Figure S5

Figure S6

Figure S7

Figure S8

Figure S9

Figure S10

Figure S11

Figure S12

Figure S13

Figure S14

Table S1-S7
