# Supplementary figures and images for: HPV-mediated nuclear export of HP1γ drives cervical tumorigenesis by downregulation of p53
Source: Cell Death Differ. 2020 Mar 23;27(9):2537–51. doi: 10.1038/s41418-020-0520-5 (PMC7429875; doi:10.1038/s41418-020-0520-5)

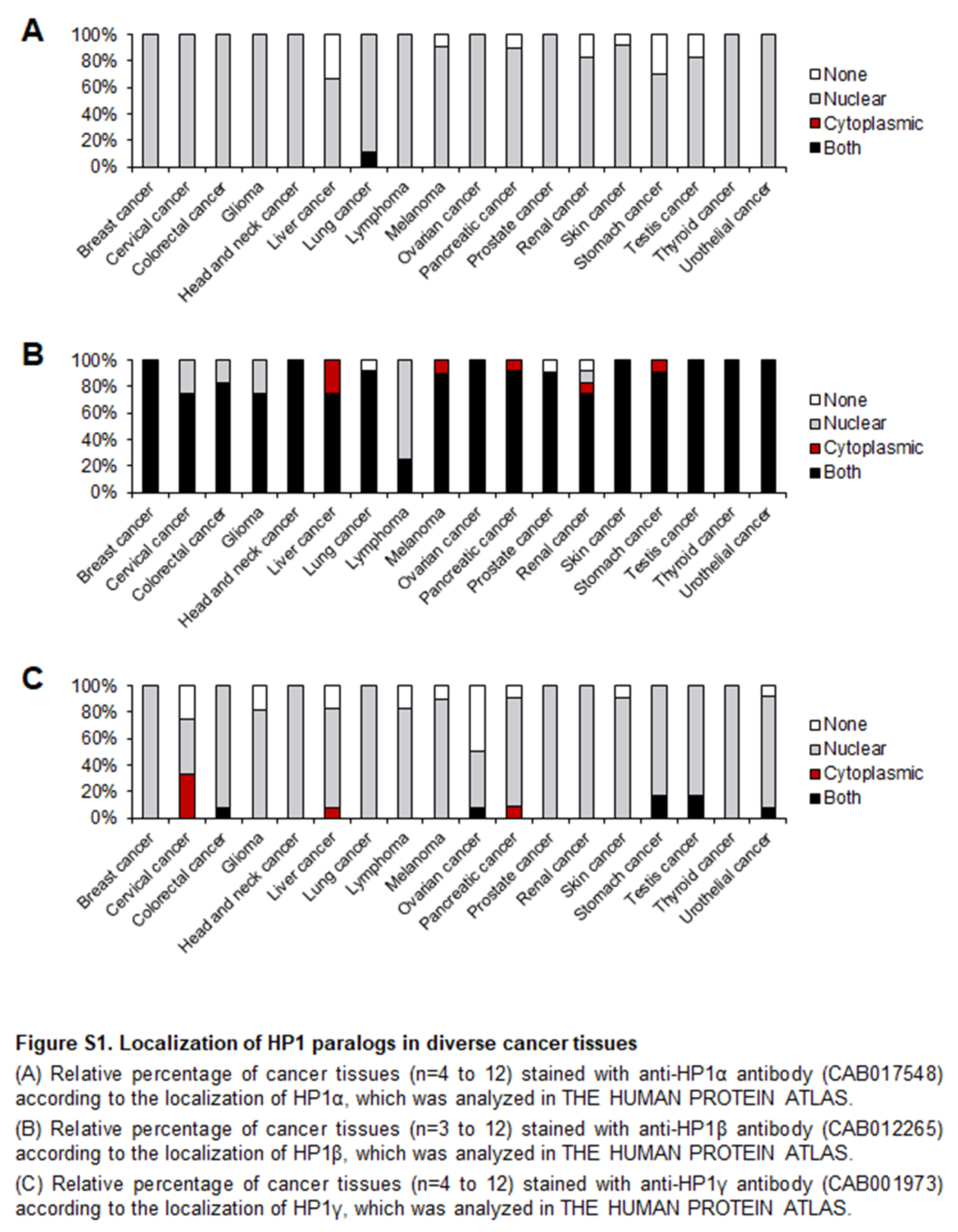

Supplement: Supplementary file 1 — Figure S1 [file 41418_2020_520_MOESM1_ESM.tif]

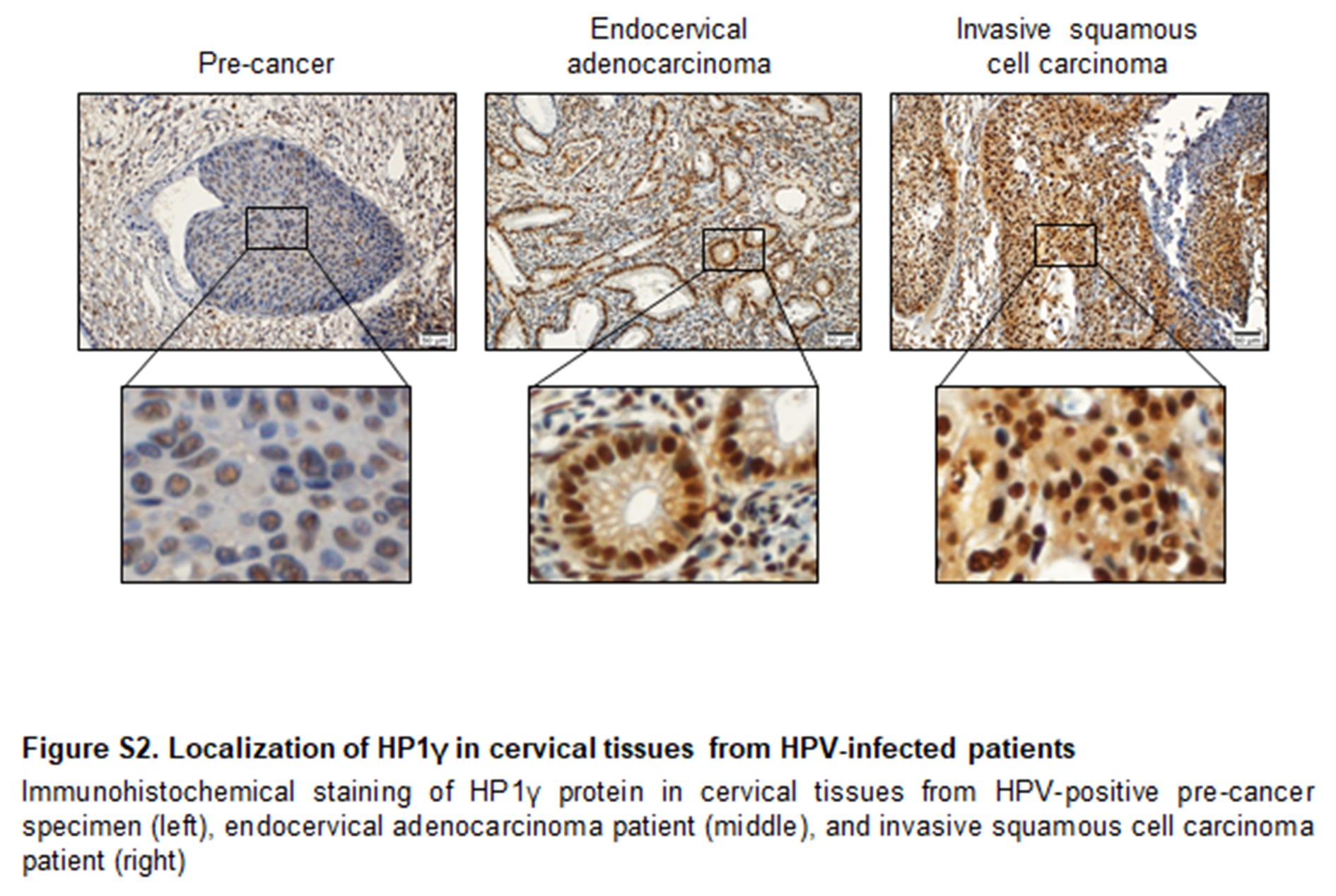

Supplement: Supplementary file 2 — Figure S2 [file 41418_2020_520_MOESM2_ESM.tif]

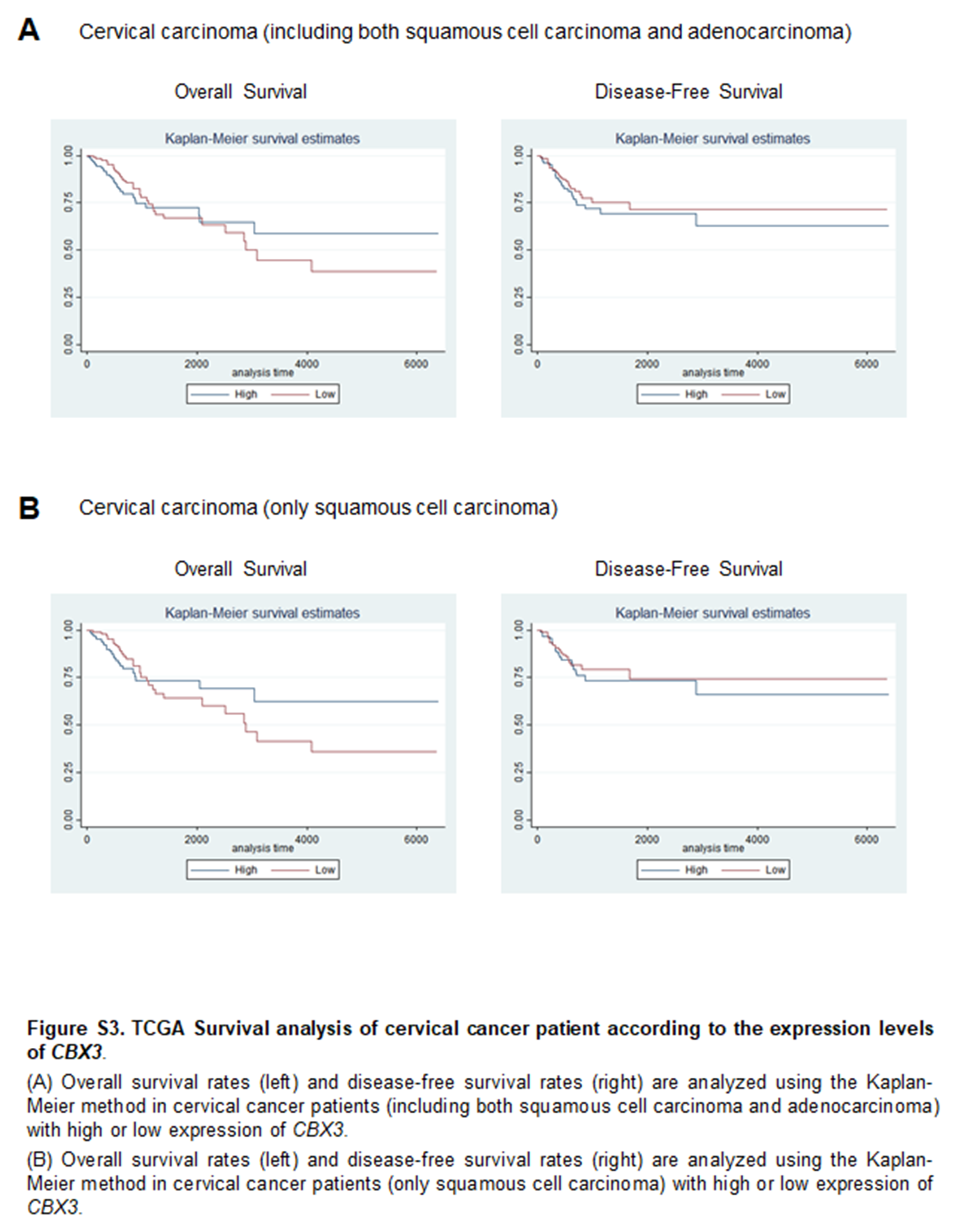

Supplement: Supplementary file 3 — Figure S3 [file 41418_2020_520_MOESM3_ESM.tif]

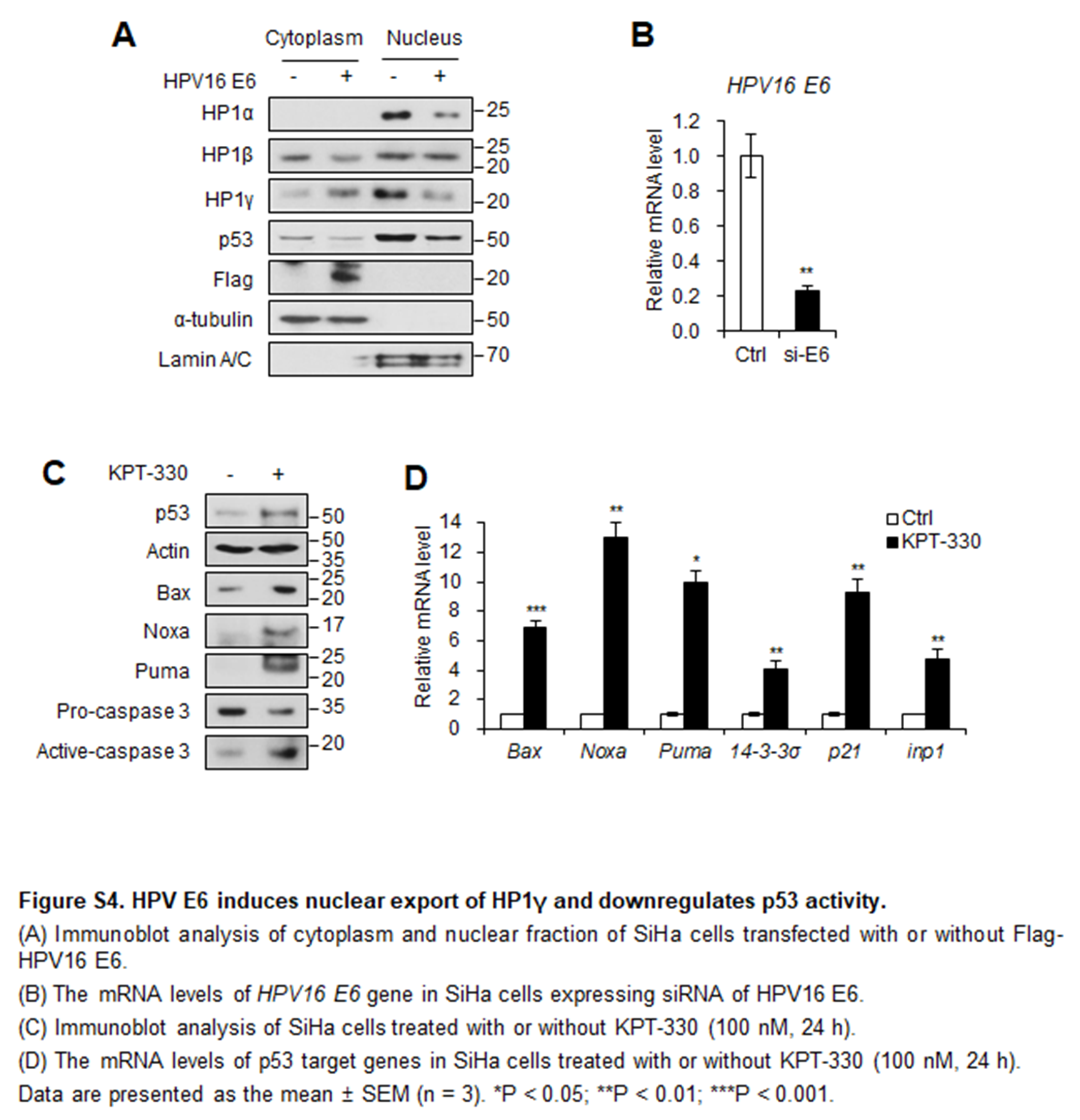

Supplement: Supplementary file 4 — Figure S4 [file 41418_2020_520_MOESM4_ESM.tif]

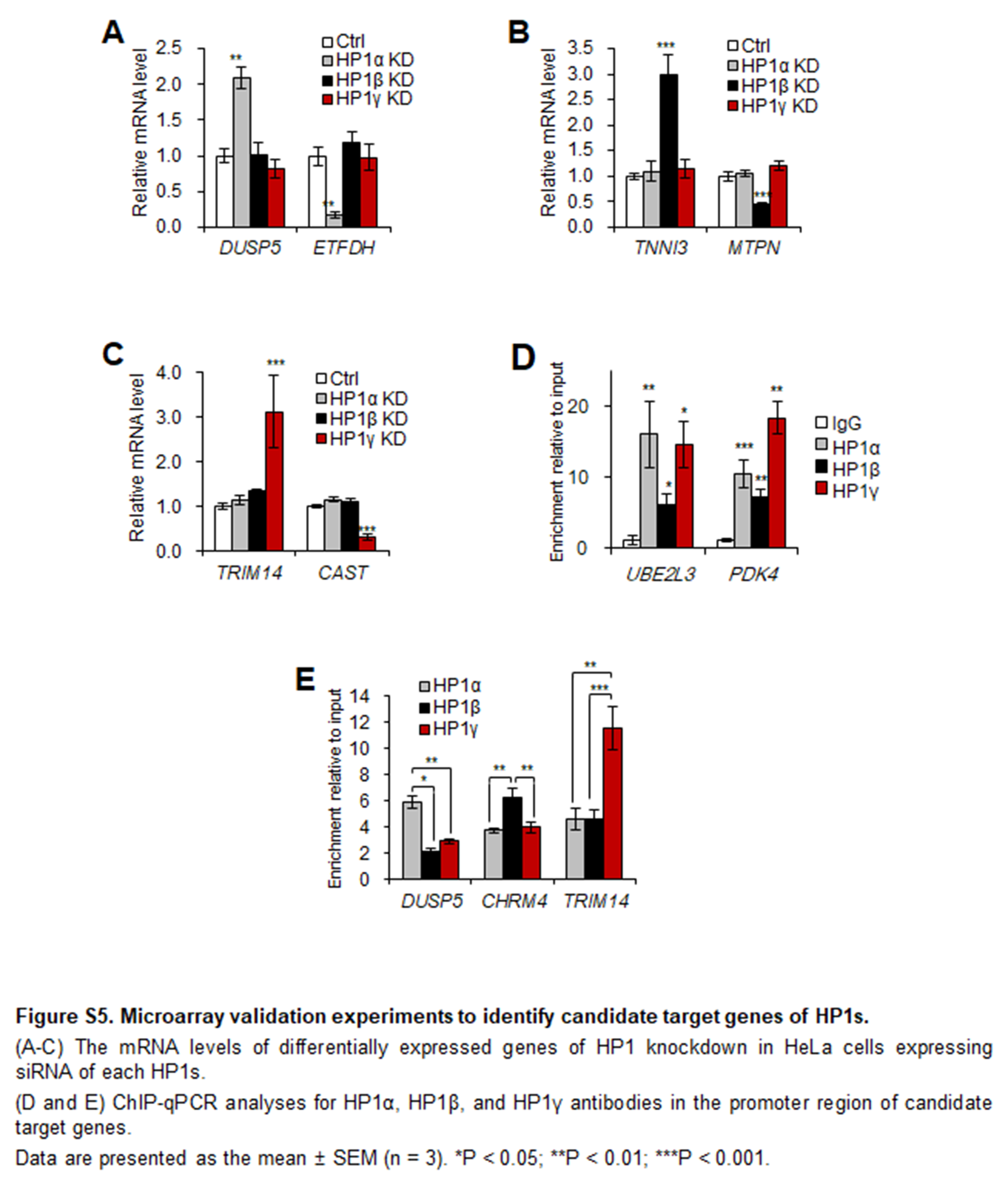

Supplement: Supplementary file 5 — Figure S5 [file 41418_2020_520_MOESM5_ESM.tif]

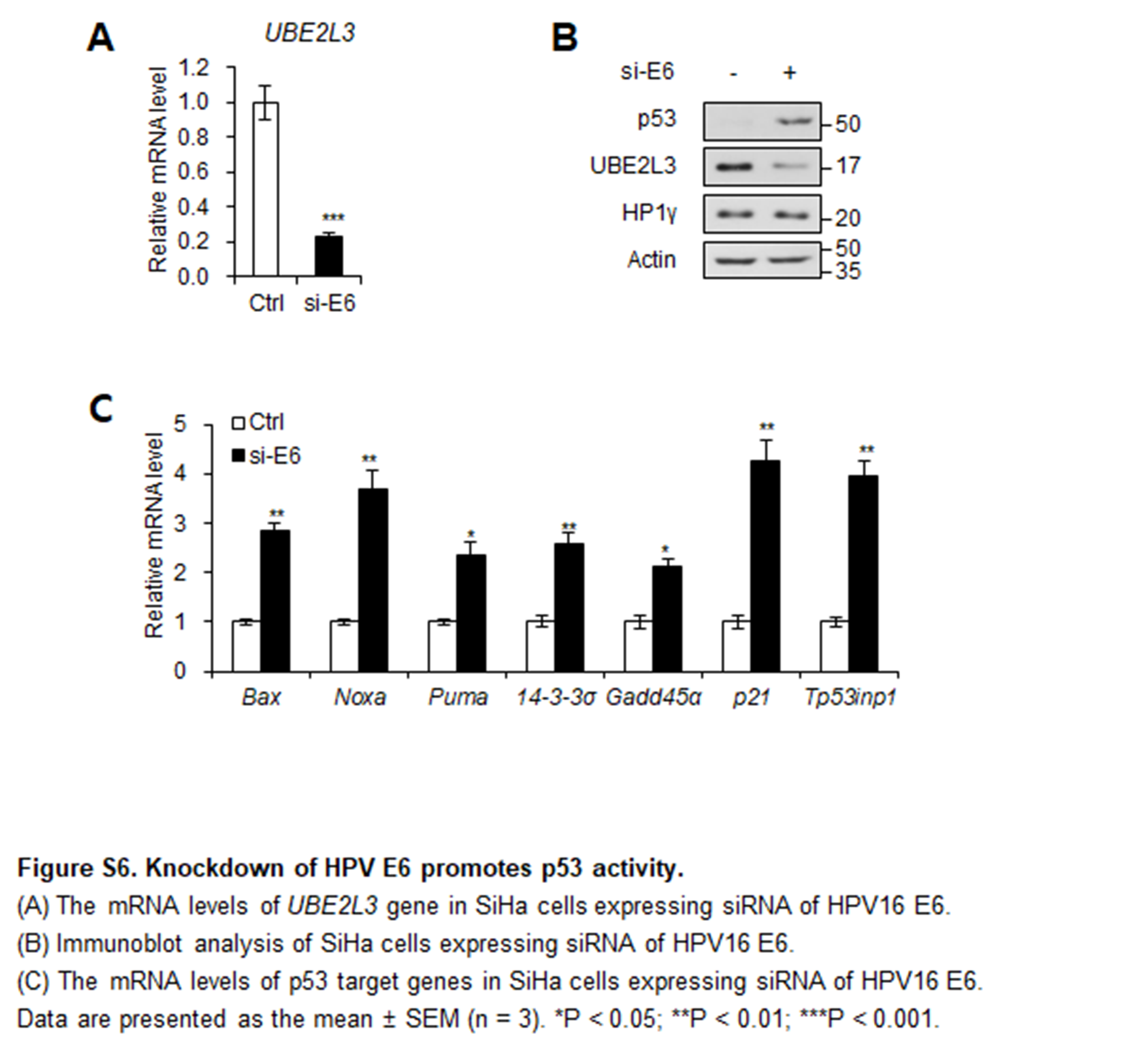

Supplement: Supplementary file 6 — Figure S6 [file 41418_2020_520_MOESM6_ESM.tif]

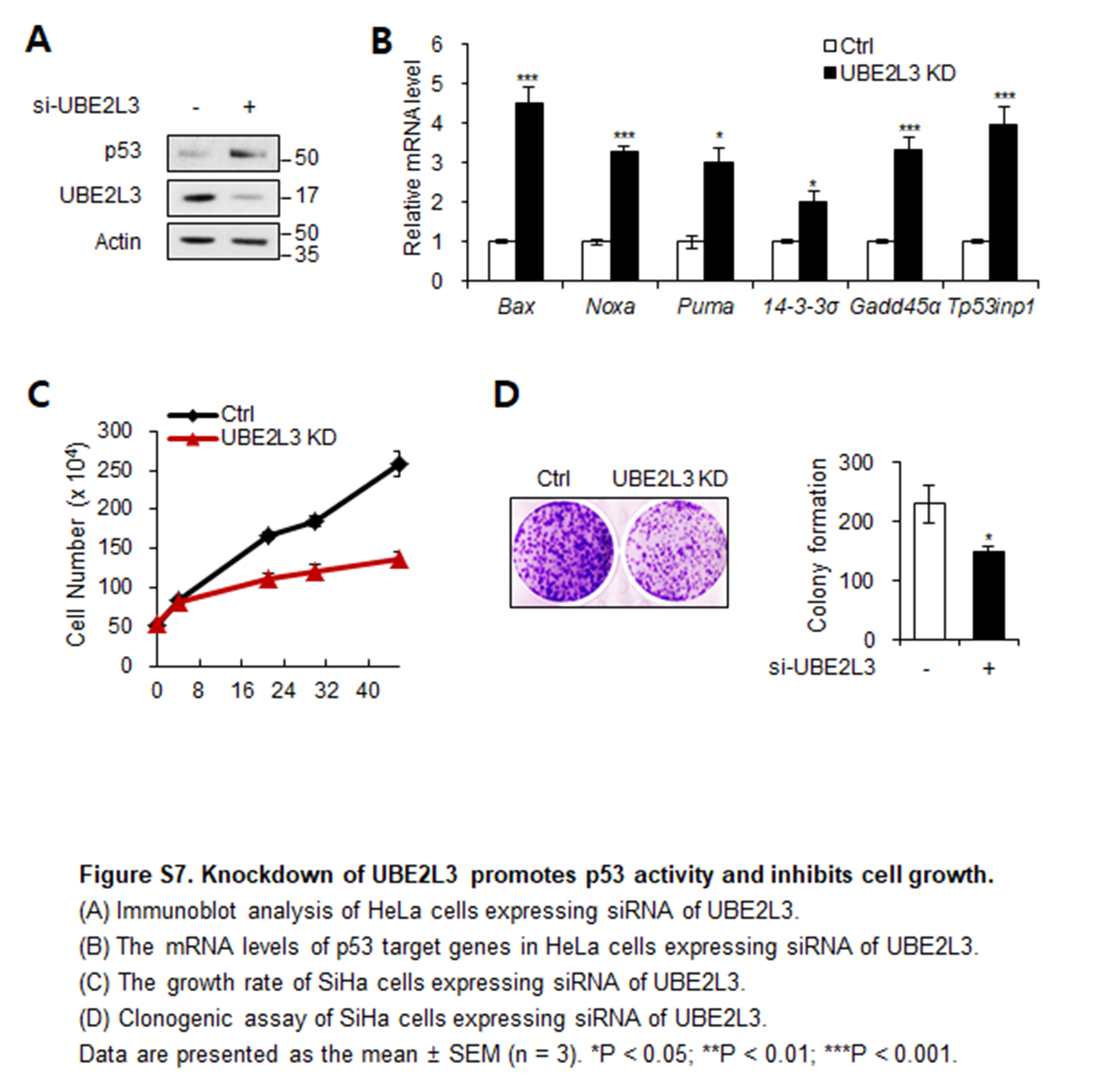

Supplement: Supplementary file 7 — Figure S7 [file 41418_2020_520_MOESM7_ESM.tif]

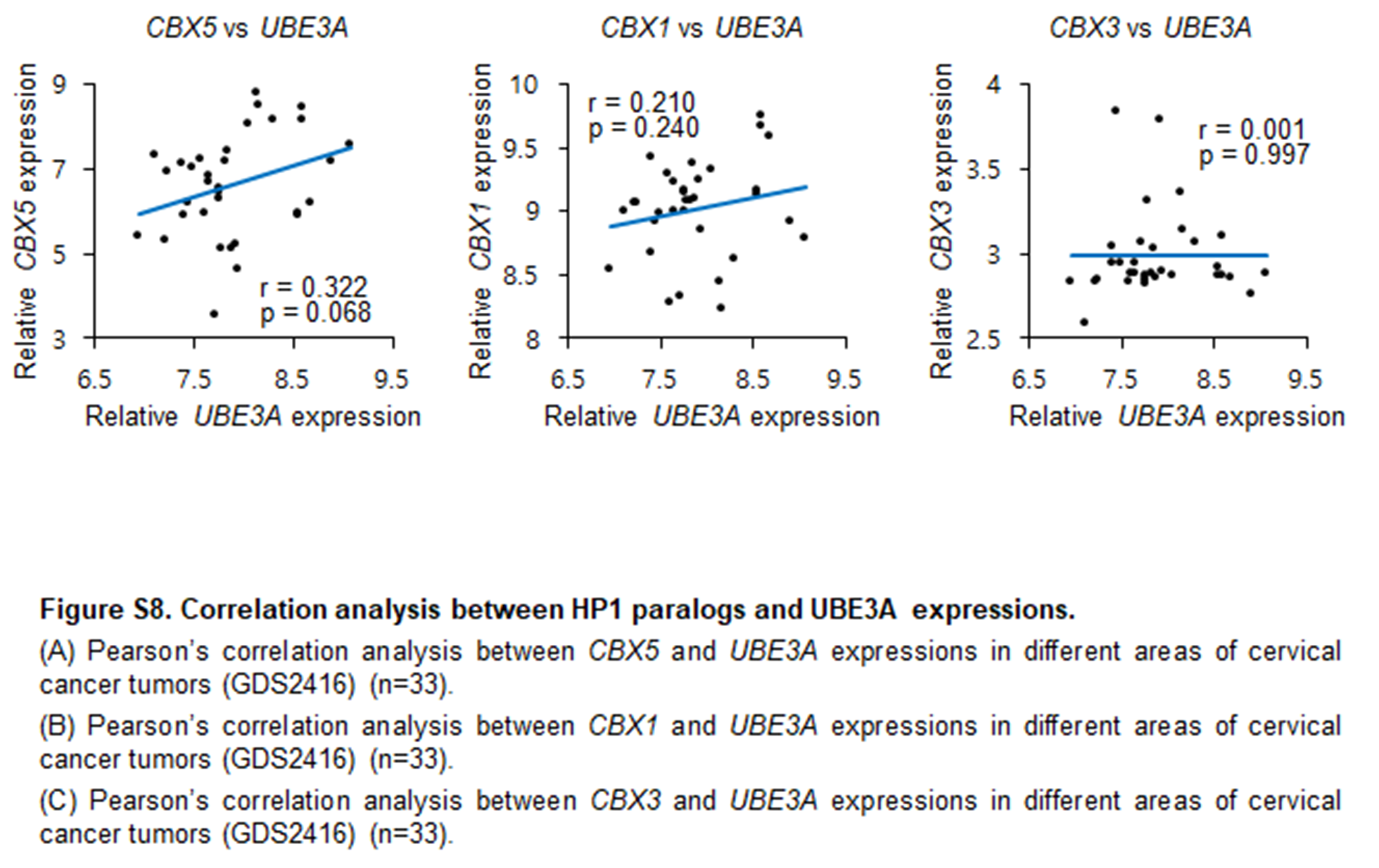

Supplement: Supplementary file 8 — Figure S8 [file 41418_2020_520_MOESM8_ESM.tif]

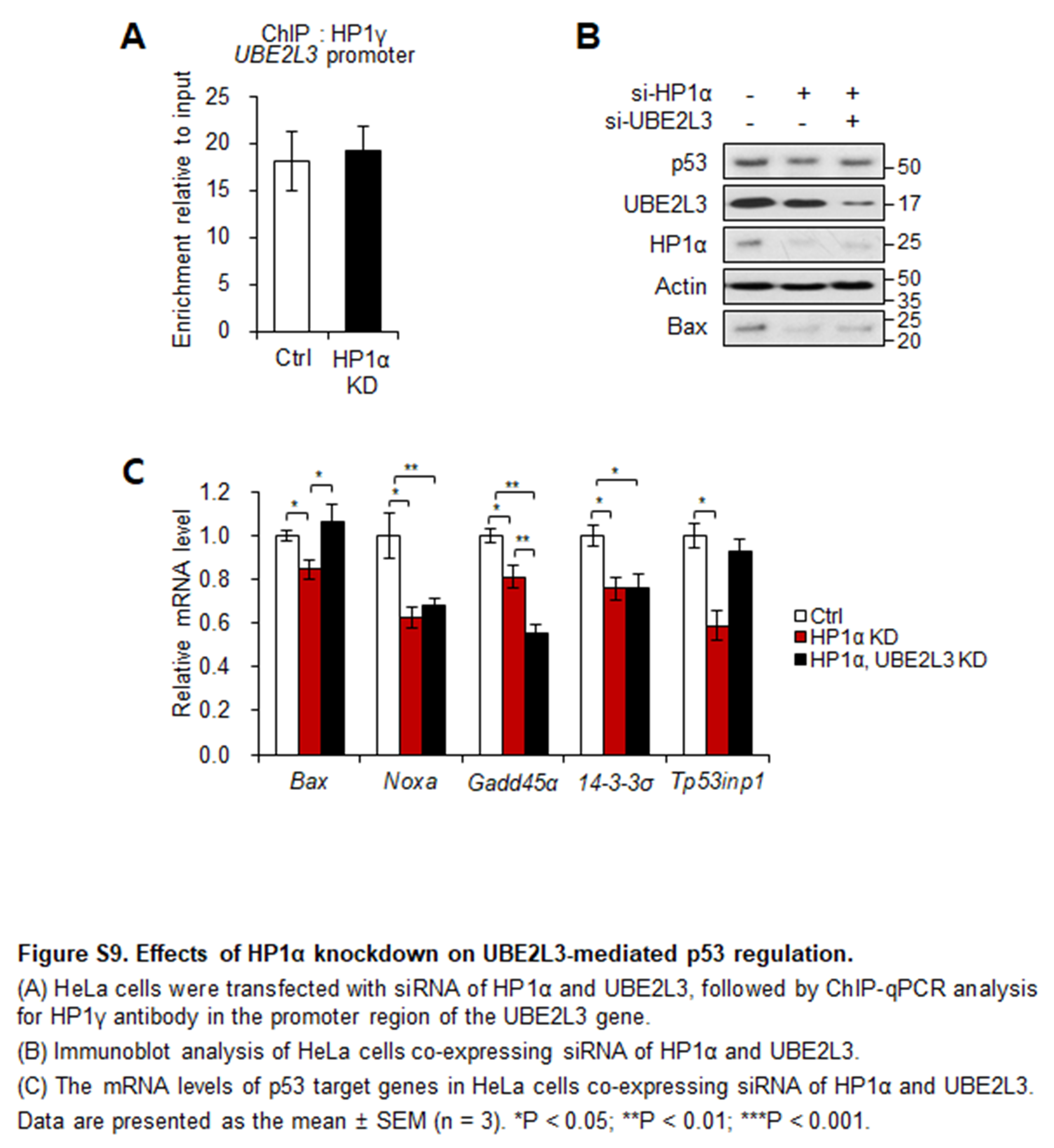

Supplement: Supplementary file 9 — Figure S9 [file 41418_2020_520_MOESM9_ESM.tif]

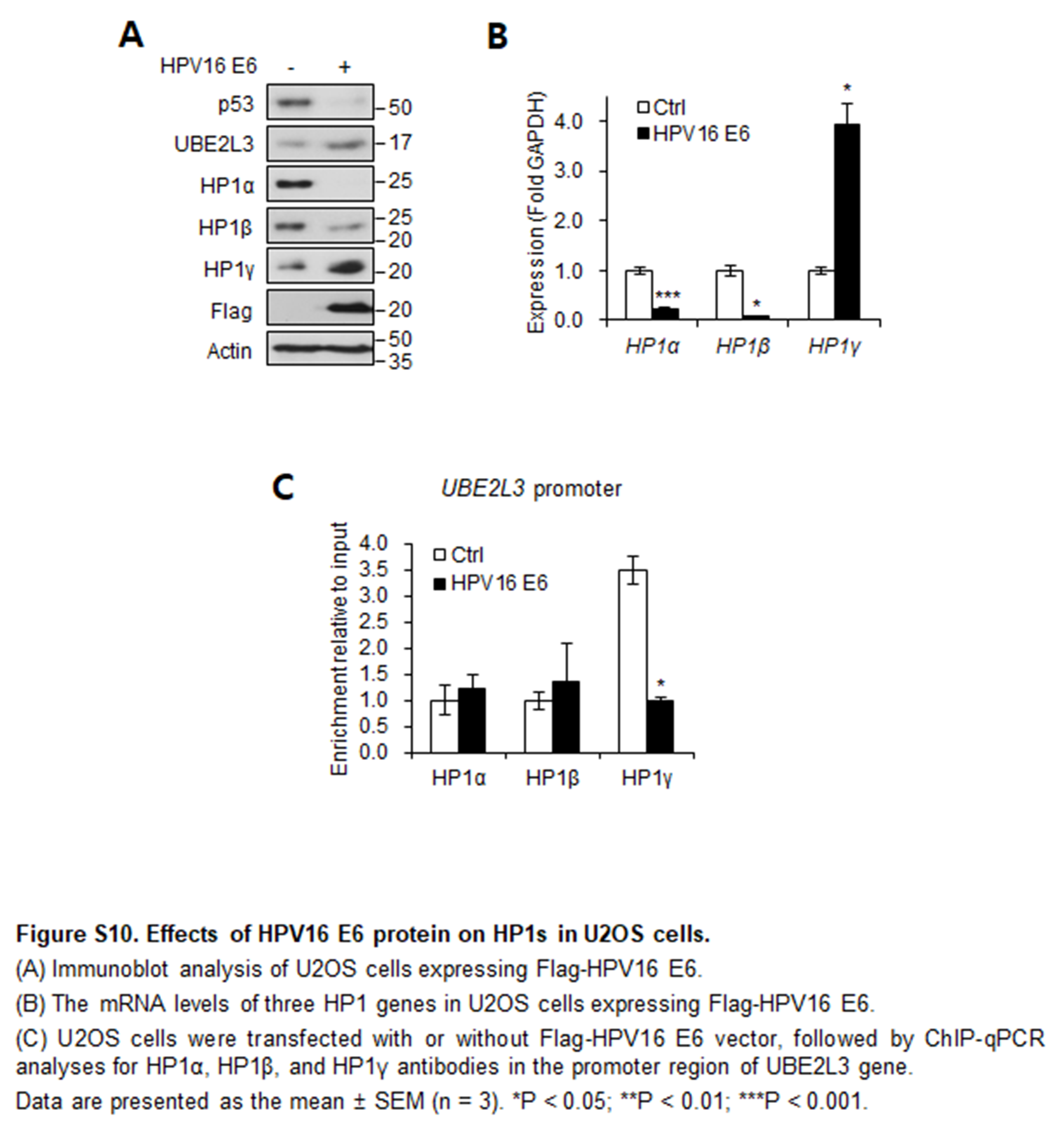

Supplement: Supplementary file 10 — Figure S10 [file 41418_2020_520_MOESM10_ESM.tif]

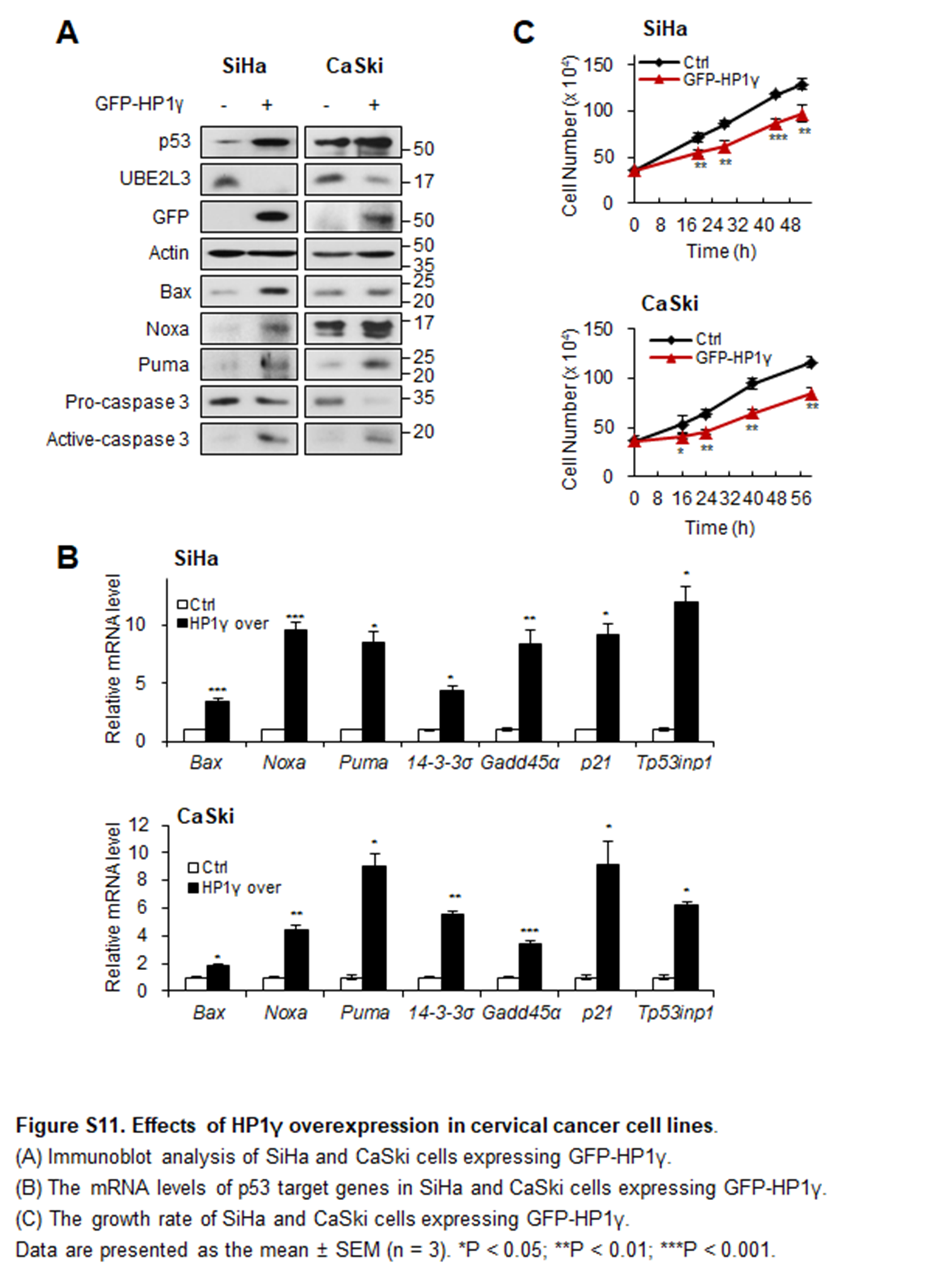

Supplement: Supplementary file 11 — Figure S11 [file 41418_2020_520_MOESM11_ESM.tif]

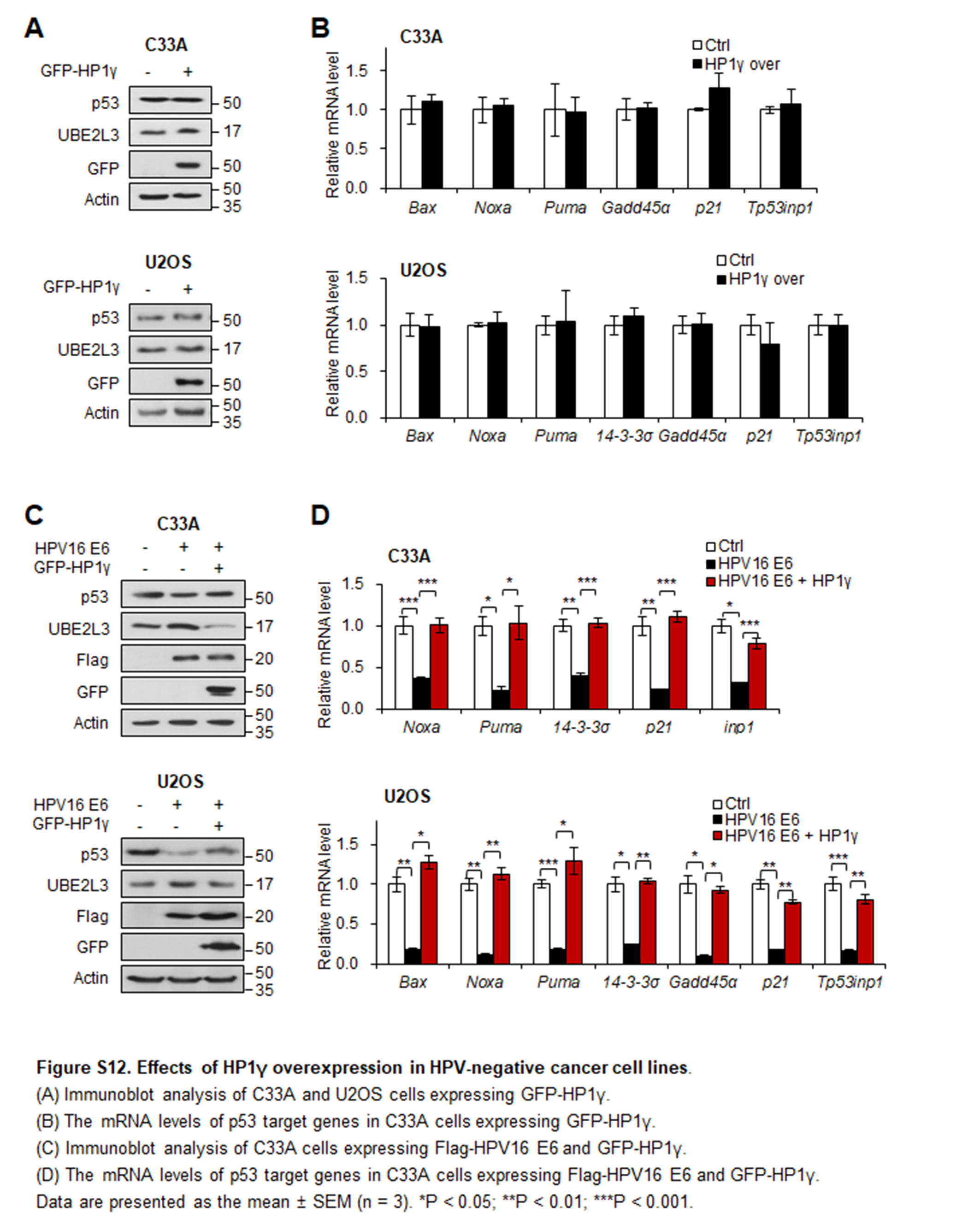

Supplement: Supplementary file 12 — Figure S12 [file 41418_2020_520_MOESM12_ESM.tif]

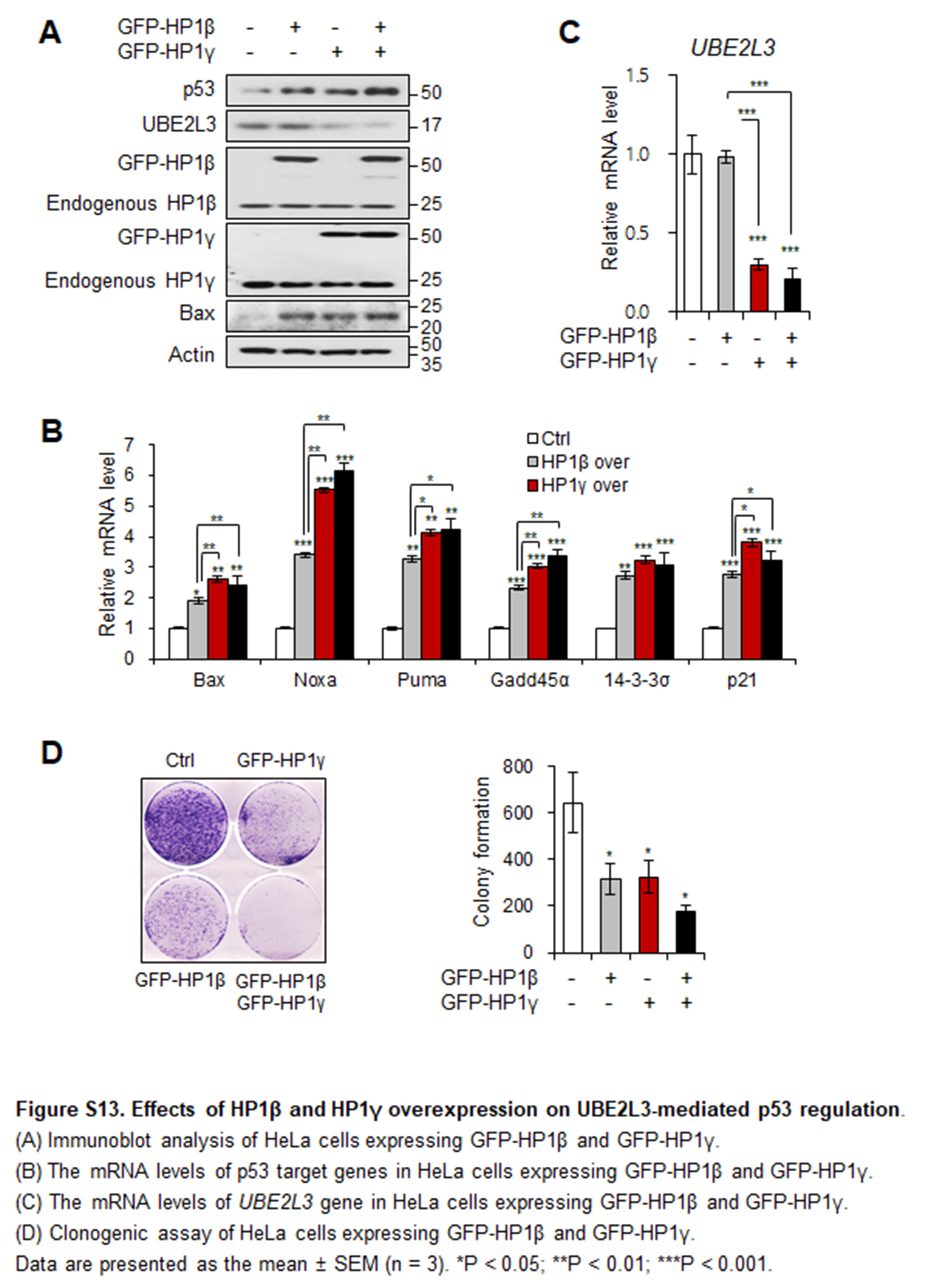

Supplement: Supplementary file 13 — Figure S13 [file 41418_2020_520_MOESM13_ESM.tif]

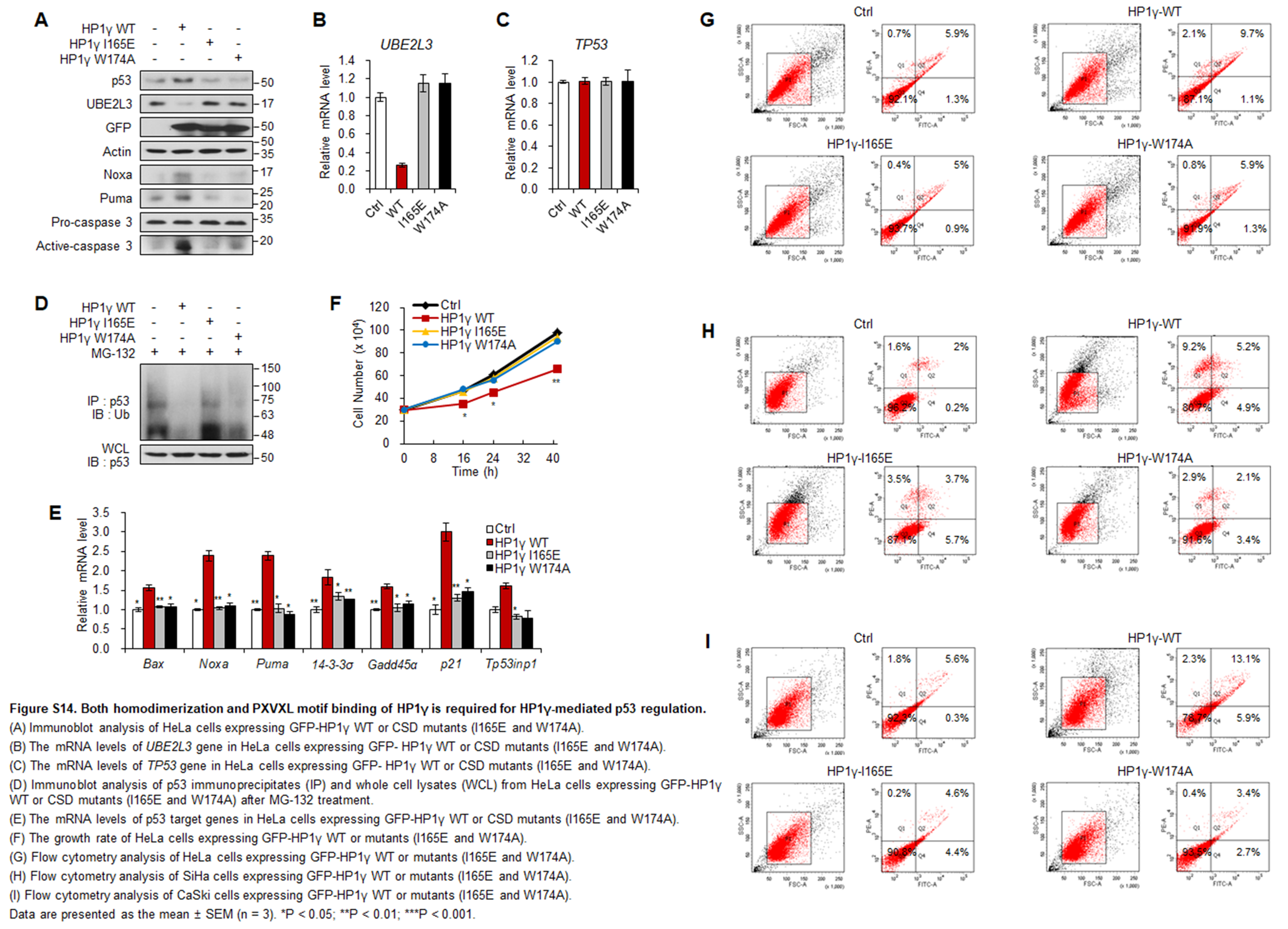

Supplement: Supplementary file 14 — Figure S14 [file 41418_2020_520_MOESM14_ESM.tif]
