# Supplementary material for: HPV-mediated nuclear export of HP1γ drives cervical tumorigenesis by downregulation of p53
Source: Cell Death Differ. 2020 Mar 23;27(9):2537–51. doi: 10.1038/s41418-020-0520-5 (PMC7429875; doi:10.1038/s41418-020-0520-5)
Supplement: Supplementary file 15 — Table S1-S7 [file 41418_2020_520_MOESM15_ESM.pdf]

Table S1. Analysis of common genes with altered by knockdown of three HP1 proteins in HeLa cells

Accession No.	Gene symbol	Gene name	Fold change		
			HP1α KD	HP1β KD	HP1γ KD
Induced					
NM_001190839	MGP	Matrix Gla protein	9.96	6.22	8.83
NR_028272	NEAT1	Nuclear paraspeckle assembly transcript 1	8.15	7.74	3.98
NM_002612	PDK4	Pyruvate dehydrogenase kinase, isozyme 4	6.77	4.26	4.00
NM_004864	GDF15	Growth differentiation factor 15	6.49	4.90	6.68
NM_194312	ESPNL	Espin-like	5.94	5.86	5.00
NM_025217	ULBP2	UL16 binding protein 2	5.53	7.38	6.40
NM_001165958	GSDMB	Gasdermin B	5.51	6.15	4.93
NM_001387	DPYSL3	Dihydropyrimidinase-like 3	5.26	6.59	3.67
NM_003641	IFITM1	Interferon induced transmembrane protein 1	5.19	3.28	3.24
NM_001831	CLU	Clusterin	4.43	2.78	3.72
NM_004089	TSC22D3	TSC22 domain family, member 3	4.39	3.18	3.70
NM_006472	TXNIP	Thioredoxin interacting protein	4.20	5.97	6.00
NM_032865	TNS4	Tensin 4	3.96	2.90	2.94
NM_002970	SAT1	Spermidine/spermine N1-acetyltransferase 1	3.89	5.03	4.13
NM_002773	PRSS8	Protease, serine, 8	3.89	4.47	5.15
.	LOC642776	Uncharacterized LOC642776	3.82	4.11	3.89
NM_152504	C20orf196	Chromosome 20 open reading frame 196	3.76	3.95	3.85
.	PARVB	Parvin, beta	3.61	3.95	4.16
NM_001099220	ZNF862	Zinc finger protein 862	3.54	4.41	4.65
NM_144717	IL20RB	Interleukin 20 receptor beta	3.53	4.79	8.54
NM_001167676	LOC100128071	uncharacterized LOC100128071	3.52	3.49	3.80
NM_001017402	LAMB3	Laminin, beta 3	3.03	2.69	2.77
NM_001040438	C6orf48	Chromosome 6 open reading frame 48	3.01	3.52	2.84
NM_001080487	PABPN1L	Poly(A) binding protein, nuclear 1-like	2.98	2.76	3.28
NR_037665	LOC100506548	Uncharacterized LOC100506548	2.98	2.91	4.95
NM_003347	UBE2L3	Ubiquitin-conjugating enzyme E2L 3	2.96	2.80	2.66
NM_001642	APLP2	Amyloid beta (A4) precursor-like protein 2	2.96	2.60	2.47
Repressed					
MGC11082	MGC11082	uncharacterized LOC84777	3.42	3.83	4.01
FBLIM1	FBLIM1	filamin binding LIM protein 1	4.04	4.10	3.69

Table S2. Top 5 genes whose transcript levels were uniquely up- or down-regulated in the HP1α KD samples.

Accession No.	Gene symbol	Gene name	Fold change	P-value
Induced				
NM_173059	ZAN	Zonadhesin	9.496364	4.659150E-09
NM_001734	C1S	Complement component 1, s subcomponent	8.331107	2.974568E-11
NM_001733	C1R	Complement component 1, r subcomponent	7.476279	2.285810E-06
NM_020801	ARRDC3	Arrestin domain containing 3	5.86302	0.0004169543
NM_004419	DUSP5	Dual specificity phosphatase 5	5.504128	0.00007968993
Repressed				
NM_004453	ETFDH	Electron-transferring-flavoprotein dehydrogenase	7.318427	0.0000002748870
NR_004388	SCARNA14	Small Cajal body-specific RNA 14	6.948727	7.594718E-07
NR_002981	SNORA51	Small nucleolar RNA, H/ACA box 51	4.71982	0.0004169543
NR_003012	SCARNA11	Small Cajal body-specific RNA 11	4.689522	0.0006263367
NM_003509	HIST1H2AI	Histone cluster 1, H2ai	4.485178	0.0006263367

Table S3. Top 5 genes whose transcript levels were uniquely up- or down-regulated in the HP1β KD samples.

Accession No.	Gene symbol	Gene name	Fold change	P-value
Induced				
NM_000363	TNNI3	Troponin I type 3 (cardiac)	16.259097	6.993954E-10
NM_000193	SHH	Sonic hedgehog	8.493775	5.577316E-12
NM_000741	CHRM4	Cholinergic receptor, muscarinic 4	7.500598	0.0001877815
NM_002283	KRT85	Keratin 85	7.368808	1.593518E-11
NR_015417	LOC284276	Uncharacterized LOC284276	6.219913	0.0004021383
Repressed				
NM_000296	PKD1	Polycystic kidney disease 1 (autosomal dominant)	6.264576	0.0001538190
NM_145808	MTPN	Myotrophin	7.074359	0.00003155635
NM_016231	NLK	Nemo-like kinase	5.270558	0.001182673
NR_002976	SNORA44	Small nucleolar RNA, H/ACA box 44	4.8549	0.003383891
NM_014551	NCAPH2	Non-SMC condensin II complex, subunit H2	4.641252	0.005612473

Table S4. Top 5 genes whose transcript levels were uniquely up- or down-regulated in the HP1γ KD samples.

Accession No.	Gene symbol	Gene name	Fold change	P-value
Induced				
NM_033219	TRIM14	Tripartite motif containing 14	23.067749	0
NM_152453	TMCO5A	Transmembrane and coiled-coil domains 5A	19.617327	5.577316E-12
NM_022834	VWA1	Von Willebrand factor A domain containing 1	12.101663	1.795037E-08
NM_001195520	LOC100507055	Uncharacterized LOC100507055	11.177078	8.031930E-08
	SYT12	Synaptotagmin XII	6.137083	0.0002084851
NM_001175	ARHGDIB	Rho GDP dissociation inhibitor (GDI) beta	5.94771	0.0004603300
Repressed				
NM_001042440	CAST	Calpastatin	5.095645	0.0003842651
NM_016206	VGLL3	Vestigial like 3 (Drosophila)	4.061235	0.01213090
	CLYBL	Citrate lyase beta like	3.898999	0.01034741
NM_178332	GNRH2	Gonadotropin-releasing hormone 2	3.786035	0.01107496
NR_004384	SNAR-F	Small ILF3/NF90-associated RNA F	3.726494	2.653404E-09
NR_024244	SNAR-G2	Small ILF3/NF90-associated RNA G2	3.479321	1.742028E-08

Table S5. Genes differentially changed (≥ 2.0 fold & p < 0.05) by knockdown of HP1α in HeLa cells.

No.	Gene Symbol	Genbank	UniGene	HP1α KD	
				Fold change	p-value
1	IFI27	NM_005532	Hs.532634	26.859403	0
2	ANGPTL4	NM_139314	Hs.9613	10.521408	1.29E-07
3	MGP	NM_001190839	Hs.365706	9.957603	7.91E-08
4	SERPINE2	NM_006216	Hs.38449	9.581943	1.19E-09
5	ZAN	NM_173059	Hs.307004	9.496364	4.66E-09
6	ABCC3	NM_003786	Hs.463421	8.817099	1.47E-07
7	C1S	NM_001734	Hs.458355	8.331107	2.97E-11
8	NEAT1	NR_028272	Hs.523789	8.153115	8.37E-12
9	SCARA5	NM_173833	Hs.591833	7.581325	3.16E-08
10	C1R	NM_001733	Hs.524224	7.476279	2.29E-06
11	C3	NM_000064	Hs.529053	7.289494	4.66E-09
12	PDK4	NM_002612	Hs.8364	6.772371	7.59E-09
13	GDF15	NM_004864	Hs.616962	6.491711	4.42E-10
14	ESPNL	NM_194312	Hs.127724	5.93671	1.32E-07
15	ARRDC3	NM_020801	Hs.24684	5.86302	0.000416954
16	ULBP2	NM_025217	Hs.656778	5.532086	0.000235748
17	GSDMB	NM_001165958	Hs.306777	5.507131	0.000753296
18	DUSP5	NM_004419	Hs.2128	5.504128	7.97E-05
19	SAA2	NM_030754	Hs.1955	5.457542	0.000694865
20	DPYSL3	NM_001387	Hs.519659	5.258794	1.04E-07
21	CCL5	NM_002985	Hs.514821	5.224349	5.76E-05
22	FOLR3	NM_000804	Hs.352	5.223196	0.000104426
23	ZFP36	NM_003407	Hs.534052	5.196503	6.30E-05
24	IFITM1	NM_003641	Hs.458414	5.194341	6.57E-06
25	STRA6	NM_001199042	Hs.24553	5.053741	0.000323547
26	SPOCK1	NM_004598	Hs.596136	5.037977	0.001609792
27	FGL1	NM_201553	Hs.491143	5.026677	0.00200383
28	ITPRIP	NM_033397	Hs.523252	5.010605	4.75E-06
29	INE1	NR_024616	Hs.657350	4.915372	0.001609792
30	RETN	NM_020415	Hs.283091	4.830264	0.00024148
31	BIRC3	NM_001165	Hs.127799	4.798046	0.000626337
32	CEACAM1	NM_001712	Hs.512682	4.751879	0.003947898
33	MAFB	NM_005461	Hs.169487	4.585422	3.21E-05
34	TFPI	NM_006287	Hs.516578	4.561722	0.002324681
35	ERRFI1	NM_018948	Hs.605445	4.518085	3.35E-05
36	IFI44	NM_006417	Hs.82316	4.476445	0.000617492
37	SAA1	NM_000331	Hs.632144	4.462091	0.000147853
38	CLU	NM_001831	Hs.436657	4.43325	4.60E-05
39	GPNMB	NM_001005340	Hs.190495	4.400399	0.007202511
40	TSC22D3	NM_004089	Hs.522074	4.394303	4.75E-05
41	XLOC_011102	BX090735	Hs.651496	4.39374	2.81E-05
42	TIMP3	NM_000362	Hs.644633	4.33292	0.000115648
43	FOLR1	NM_016725	Hs.73769	4.303211	2.18E-05
44	S100P	NM_005980	Hs.2962	4.294888	1.50E-06
45	C1RL	NM_016546	Hs.631730	4.287228	0.008638816
46	TXNIP	NM_006472	Hs.533977	4.196566	0.000113703
47	FNDC3A	NM_001079673	Hs.508010	4.189782	0.00015986
48	XLOC_006743	BU195360	Hs.386254	4.185911	0.00335526
49	IRF9	NM_006084	Hs.1706	4.08858	0.014267871
50	LOC100507429	XR_110179	Hs.729388	4.019635	0.010825768
51	FOS	NM_005252	Hs.728079	4.001043	0.006194146
52	LOC100506694	XR_132650		3.982683	0.016302046
53	ADAMTS6	NM_197941	Hs.482291	3.978996	0.018585883
54	PGM2L1	NM_173582	Hs.26612	3.969743	0.000694865
55	TNS4	NM_032865	Hs.438292	3.959583	0.000288335
56	LAMB2	NM_002292	Hs.439726	3.95379	0.001609792
57	GADD45B	NM_015675	Hs.110571	3.950601	0.000288335
58	LOC653075	NR_033933		3.932014	0.020307586
59	LOC100507429	XR_110179	Hs.729388	3.92718	0.004914024
60	INHBA	NM_002192	Hs.583348	3.898394	0.018267071
61	SAT1	NM_002970	Hs.28491	3.894829	0.000575977
62	PRSS8	NM_002773	Hs.75799	3.886108	0.006195565
63	MATN4	NM_003833	Hs.278489	3.85507	0.000607483

64	GPR126	NM_020455	Hs.726094	3.826637	0.01190591
65	LOC642776	BC003645		3.820272	0.00588362
66	LOC100506694	XR_132650	Hs.567934	3.81519	0.0237934
67	LOC285084	NR_038897	Hs.275398	3.802646	0.013614443
68	ALCAM	NM_001627	Hs.591293	3.774765	0.028458113
69	C20orf196	NM_152504	Hs.529340	3.75742	0.000626337
70	SRGN	NM_002727	Hs.1908	3.755696	0.000626337
71	KRT17	NM_000422	Hs.2785	3.754162	0.028332299
72	LOC157627	NR_024281	Hs.12513	3.729918	0.024946285
73	LOC283731	BC050067	Hs.631696	3.727685	0.020108548
74	HERC2P2	NR_002824	Hs.728176	3.716579	0.02784158
75	XLOC_009487	BC039105	Hs.639242	3.709807	0.004450894
76	IFIT2	NM_001547	Hs.437609	3.695348	0.015914706
77	ITSN2	NM_147152	Hs.432562	3.67393	0.003477412
78	ICAM5	NM_003259	Hs.465862	3.646695	0.011262619
79	COL4A5	NM_033380	Hs.369089	3.643665	0.01181131
80	AHSA2	NM_152392	Hs.655602	3.619308	0.018267071
81	LY86	NM_004271	Hs.653138	3.617407	0.000738027
82	PARVB	AK309987	Hs.475074	3.609713	0.004954664
83	SAMD9L	NM_152703	Hs.489118	3.59826	0.023352964
84	MALAT1	NR_002819	Hs.621695	3.59085	0.013402267
85	F2R	NM_001992	Hs.482562	3.589349	0.042894017
86	XLOC_009723	XR_110375	Hs.729166	3.589057	0.001073351
87	GPR37	NM_005302	Hs.406094	3.582849	0.033218683
88	PRDM1	NM_001198	Hs.436023	3.574829	0.041126549
89	ANKH	NM_054027	Hs.156727	3.552462	0.022222844
90	CCDC68	NM_025214	Hs.120790	3.551822	0.044710219
91	ZNF862	NM_001099220	Hs.301277	3.543578	0.04829676
92	BTN2A2	NM_181531	Hs.373938	3.534401	0.049062057
93	IL20RB	NM_144717	Hs.61232	3.532761	0.020307586
94	ZDHHC11	NM_024786	Hs.729226	3.522527	0.001449949
95	LOC100128071	NM_001167676	Hs.512763	3.519633	0.01506256
96	SOCS3	NM_003955	Hs.527973	3.515215	0.005556498
97	IL6	NM_000600	Hs.654458	3.50028	0.032789006
98	DUSP1	NM_004417	Hs.171695	3.494251	0.001732785
99	OSMR	NM_001168355		3.454074	0.001720598
100	TPP1	NM_000391	Hs.523454	3.447619	0.016761725
101	HLA-A	NM_002116	Hs.181244	3.428336	0.002134385
102	SLC2A14	BC060766	Hs.655169	3.428103	0.002270919
103	HLA-C	M26429	Hs.654404	3.364393	0.000321474
104	ANG	NM_001145	Hs.593708	3.356214	0.037354925
105	HRASLS2	NM_017878	Hs.272805	3.344064	0.026881102
106	PRIC285	NM_001037335	Hs.517180	3.334435	0.017540125
107	HLA-B	NM_005514	Hs.77961	3.329784	0.001073351
108	PGCP	NM_016134	Hs.156178	3.297323	0.027295838
109	SECTM1	NM_003004	Hs.558009	3.278468	0.004446996
110	SLC2A3	NM_006931	Hs.419240	3.27781	0.025933566
111	PTPRM	NM_002845	Hs.49774	3.259424	0.037632512
112	HNRNPU-AS1	NR_026778	Hs.723308	3.252992	0.049602142
113	PON2	NM_000305	Hs.530077	3.211825	0.002134385
114	HLA-G	NM_002127	Hs.512152	3.204006	0.000892755
115	APP	NM_000484	Hs.434980	3.174544	0.005373329
116	CTSC	NM_001114173	Hs.128065	3.168297	0.006194146
117	PRPF4B	NM_003913	Hs.159014	3.166019	0.012029919
118	ICAM1	NM_000201	Hs.643447	3.158813	0.006195565
119	TGFBR3	NM_003243	Hs.482390	3.158189	0.041247081
120	ISG15	NM_005101	Hs.458485	3.156562	0.002655624
121	ARSA	NM_000487	Hs.88251	3.155684	0.006793643
122	KIAA0040	NM_001162893	Hs.518138	3.127911	0.011661515
123	P4HA2	NM_004199	Hs.519568	3.126397	0.007212025
124	HLA-J	NR_024240	Hs.720762	3.107618	0.004446996
125	GPCPD1	NM_019593	Hs.636359	3.089909	0.049062057
126	BCL6	NM_001130845	Hs.478588	3.071045	0.009966446
127	MIPEP	NM_005932	Hs.507498	3.068698	0.009937679
128	XLOC_l2_008124	XR_109954		3.060226	0.005771848
129	ATP2B4	NM_001001396	Hs.343522	3.031596	0.014256666
130	TGFBI	NM_000358	Hs.369397	3.027828	0.043421341
131	LAMB3	NM_001017402	Hs.497636	3.026477	0.011734991
132	NUCB2	NM_005013	Hs.654599	3.010934	0.031456232
133	C6orf48	NM_001040438	Hs.640836	3.008289	0.006195565

134	HLA-F	NM_018950	Hs.519972	2.986761	0.009366716
135	CTBS	NM_004388	Hs.513557	2.986548	0.032188766
136	PABPN1L	NM_001080487	Hs.730522	2.982456	0.020164956
137	LOC100506548	NR_037665		2.977961	0.013460576
138	ATP6AP2	NM_005765	Hs.495960	2.974112	0.013402267
139	TBC1D2	NM_018421	Hs.371016	2.965324	0.013460576
140	UBE2L3	NM_003347	Hs.108104	2.959352	0.004450894
141	APLP2	NM_001642	Hs.370247	2.956332	0.013312474
142	ITGB1	NM_133376	Hs.643813	2.95624	0.028458113
143	IFIT2	NM_001547	Hs.437609	2.895062	0.018078232
144	GNS	NM_002076	Hs.334534	2.892269	0.018267071
145	GRN	NM_002087	Hs.514220	2.888613	0.001609792
146	ST6GAL1	NM_173216	Hs.207459	2.887857	0.019538479
147	RGS2	NM_002923	Hs.78944	2.875388	0.024946285
148	ITM2B	NM_021999	Hs.643683	2.838012	0.022230057
149	HEXB	NM_000521	Hs.69293	2.836745	0.023352964
150	PROSER1	NM_025138	Hs.318526	2.829606	0.022766464
151	EFEMP1	NM_001039348	Hs.76224	2.826088	0.004394591
152	TIMP1	NM_003254	Hs.522632	2.794233	0.026237647
153	RHEBL1	NM_144593	Hs.159013	2.79396	0.026274314
154	KLF10	NM_005655	Hs.435001	2.793494	0.030923124
155	STOM	NM_198194	Hs.253903	2.758079	0.031118386
156	TNFSF9	NM_003811	Hs.1524	2.756763	0.030923124
157	GPR64	NM_001079858	Hs.146978	2.755676	0.04260495
158	CD109	NM_133493	Hs.399891	2.694037	0.040847918
159	PXDN	NM_012293	Hs.332197	2.683645	0.039208745
160	NPC2	NM_006432	Hs.433222	2.673816	0.031182049
161	SCARB2	NM_001204255	Hs.349656	2.644054	0.03774759
162	WISP2	NM_003881	Hs.592145	2.628981	0.030867609
163	EPAS1	NM_001430	Hs.468410	2.613268	0.02554286
164	IGFBP7	NM_001553	Hs.479808	2.578034	0.048933694
165	NNMT	NM_006169	Hs.503911	2.482633	0.025933566
166	SNAR-G1	NR_004383	Hs.621635	-2.174176	0.033944314
167	UNG	NM_003362	Hs.191334	-2.674241	0.039362741
168	XLOC_001620	DB077273	Hs.585967	-2.677844	0.044710219
169	NT5C3	NM_001002010	Hs.487933	-3.009954	0.04829676
170	MARC1	NM_022746	Hs.497816	-3.051764	0.042894017
171	SCARNA20	NR_002999		-3.087749	0.034962176
172	SNORA62	NR_002324	Hs.731290	-3.167851	0.028112936
173	PCBP2	NM_005016	Hs.546271	-3.346212	0.016967048
174	MNAT1	NM_002431	Hs.509523	-3.360528	0.017311698
175	SNORA75	NR_002921		-3.404859	0.018499713
176	MGC11082	BC094703	Hs.659053	-3.415631	0.030521967
177	DNAJC11	NM_018198	Hs.462640	-3.419319	0.015653519
178	PROP1	NM_006261	Hs.158301	-3.513219	0.012142866
179	RPS26	NM_001029	Hs.567235	-3.530925	0.014456894
180	SCARNA16	NR_003013	Hs.731291	-3.756966	0.005975635
181	SWAP70	NM_015055	Hs.153026	-3.777518	0.004954664
182	SNORA2B	NR_002951		-3.827425	0.005373329
183	MMP24	NM_006690	Hs.715494	-3.855712	0.003877239
184	FBLIM1	NM_001024215	Hs.530101	-4.044344	0.007770021
185	FSCN1	NM_003088	Hs.118400	-4.174131	3.35E-05
186	SCARNA8	NR_003009	Hs.689635	-4.204794	0.001468304
187	CBX5	NM_001127322	Hs.349283	-4.47348	0.000626337
188	HIST1H2AI	NM_003509	Hs.534035	-4.485178	0.000626337
189	SCARNA11	NR_003012	Hs.689692	-4.689522	0.000626337
190	SNORA51	NR_002981	Hs.689698	-4.71982	0.000416954
191	SNORA67	NR_002912		-4.760481	0.000107323
192	MEST	NM_002402	Hs.270978	-4.798696	0.000246083
193	SNORA79	NR_003021	Hs.693251	-4.848044	0.00024148
194	SCARNA13	NR_003002	Hs.728662	-4.94873	0.000185116
195	SNORA44	NR_002976	Hs.730337	-6.254077	6.57E-06
196	SCARNA14	NR_004388		-6.948727	7.59E-07
197	ETFDH	NM_004453	Hs.155729	-7.318427	2.75E-07

Table S6. Genes differentially changed (≥ 2.0 fold & $p < 0.05$) by knockdown of HP1β in HeLa cells.

No.	Gene Symbol	Genbank	UniGene	HP1β KD	
				Fold change	p-value
1	TNNI3	NM_000363	Hs.709179	16.259097	6.99E-10
2	IFI27	NM_005532	Hs.532634	14.230864	1.12E-11
3	SERPINE2	NM_006216	Hs.38449	11.200694	0
4	SHH	NM_000193	Hs.164537	8.493775	5.58E-12
5	NEAT1	NR_028272	Hs.523789	7.741641	2.79E-12
6	CHRM4	NM_000741	Hs.248100	7.500598	0.000187782
7	ULBP2	NM_025217	Hs.656778	7.397819	1.47E-06
8	KRT85	NM_002283	Hs.182507	7.368808	1.59E-11
9	XLOC_012826	AK309717	Hs.639087	7.15515	2.29E-11
10	DPYSL3	NM_001387	Hs.519659	6.591823	1.67E-11
11	INHBA	NM_002192	Hs.583348	6.581131	0.000431249
12	XLOC_001048	DA336930	Hs.213122	6.432308	0.001322555
13	LOC284276	NR_015417	Hs.390287	6.219913	0.000402138
14	MGP	NM_001190839	Hs.365706	6.216354	0.00065269
15	GSDMB	NM_001165958	Hs.306777	6.149816	0.002092091
16	TFPI	NM_006287	Hs.516578	6.129642	0.000162454
17	TXNIP	NM_006472	Hs.533977	5.972984	4.98E-09
18	RETN	NM_020415	Hs.283091	5.898392	4.94E-07
19	ANGPTL4	NM_139314	Hs.9613	5.890551	0.003057411
20	ESPNL	NM_194312	Hs.127724	5.862545	6.74E-09
21	SPOCK1	NM_004598	Hs.596136	5.761638	0.00267171
22	LOC643837	NR_015368	Hs.720079	5.547388	2.12E-08
23	MATN4	NM_003833	Hs.278489	5.47965	2.67E-08
24	XLOC_011102	BX090735	Hs.651496	5.142891	1.37E-07
25	SAT1	NM_002970	Hs.28491	5.034792	2.02E-07
26	SEPP1	NM_005410	Hs.730647	5.024703	0.011144942
27	MMP12	NM_002426	Hs.1695	4.96516	0.012788696
28	GDF15	NM_004864	Hs.616962	4.898854	1.35E-07
29	FOLR3	NM_000804	Hs.352	4.865102	0.000187782
30	LOC283731	BC050067	Hs.631696	4.830188	0.007938025
31	IL20RB	NM_144717	Hs.61232	4.785551	0.00224412
32	FAM154A	NM_153707	Hs.98943	4.698905	0.019550791
33	ADAMTS6	NM_197941	Hs.482291	4.558831	0.025789897
34	ABCC3	NM_003786	Hs.463421	4.495607	0.013250369
35	PRSS8	NM_002773	Hs.75799	4.471912	0.002269155
36	HNRNPU-AS1	NR_026778	Hs.723308	4.446142	0.010246017
37	XLOC_009487	BC039105	Hs.639242	4.421816	5.71E-05
38	ZNF862	NM_001099220	Hs.301277	4.412078	0.031331252
39	FNDC3A	NM_001079673	Hs.508010	4.334949	6.48E-06
40	LOC285084	NR_038897	Hs.275398	4.291218	0.016483678
41	PDK4	NM_002612	Hs.8364	4.261141	9.91E-06
42	LY86	NM_004271	Hs.653138	4.201446	1.54E-05
43	LOC157627	NR_024281	Hs.12513	4.200722	0.03656229
44	LOC100129119	XR_109159		4.110803	0.029201955
45	LOC642776	BC003645		4.10686	0.003057411
46	PLA2G2F	NM_022819	Hs.302034	4.056873	0.044505528
47	PARVB	AK309987	Hs.475074	3.951414	0.000313769
48	C20orf196	NM_152504	Hs.529340	3.950205	8.24E-05
49	ICAM5	NM_003259	Hs.465862	3.726095	0.018682908
50	MIPEP	NM_005932	Hs.507498	3.597612	0.000430055
51	FOLR1	NM_016725	Hs.73769	3.597028	0.000260709
52	COL4A5	NM_033380	Hs.369089	3.53811	0.037190267
53	C6orf48	NM_001040438	Hs.640836	3.517077	0.000267435
54	LOC100128071	NM_001167676	Hs.512763	3.488655	0.035748738
55	LOC645645	AK126090	Hs.159711	3.442476	0.026813013
56	RECK	NM_021111	Hs.728961	3.43285	0.039990782
57	ZFP36	NM_003407	Hs.534052	3.397385	0.03101652
58	PGM2L1	NM_173582	Hs.26612	3.360988	0.00289836
59	VASN	NM_138440	Hs.372579	3.341534	0.001143144
60	C3	NM_000064	Hs.529053	3.332362	0.010246017
61	C5orf43	NM_001048249	Hs.508479	3.318328	0.001737154
62	IFITM1	NM_003641	Hs.458414	3.280047	0.006966407

63	XLOC_l2_008124	XR_109954		3.237759	0.001459417
64	B3GALNT1	NM_001038628	Hs.418062	3.218532	0.003697761
65	CYP1B1	NM_000104	Hs.154654	3.194328	0.002646966
66	TSC22D3	NM_004089	Hs.522074	3.178927	0.003057411
67	C9orf5	NM_032012	Hs.308074	3.143948	0.002297032
68	SCARNA17	NR_003003	Hs.668351	3.133057	0.004094828
69	ICAM1	NM_000201	Hs.643447	3.129285	0.002714312
70	DKK1	NM_012242	Hs.40499	3.088397	0.014386831
71	ATRNL1	NM_207303	Hs.501127	2.990866	0.004592395
72	PDIA5	NM_006810	Hs.477352	2.965867	0.013250369
73	ITM2B	NM_021999	Hs.643683	2.95679	0.007228055
74	F11R	NM_016946	Hs.517293	2.953758	0.007938025
75	FKBP14	NM_017946	Hs.571333	2.952697	0.039662262
76	HIST1H2BK	NM_080593	Hs.437275	2.943623	0.008756471
77	RBKS	NM_022128	Hs.11916	2.935349	0.013419873
78	APP	NM_000484	Hs.434980	2.919011	0.009796372
79	RN5-8S1	NR_003285	Hs.631413	2.911031	0.005423394
80	LOC100506548	NR_037665		2.910781	0.006966407
81	TNS4	NM_032865	Hs.438292	2.901978	0.008107039
82	TMEM71	NM_144649	Hs.293842	2.901005	0.015946216
83	ANXA1	NM_000700	Hs.494173	2.876358	0.010536908
84	PRPF4B	NM_003913	Hs.159014	2.864938	0.017549799
85	GNS	NM_002076	Hs.334534	2.86258	0.013034239
86	ATP6AP2	NM_005765	Hs.495960	2.858949	0.010536908
87	XLOC_011837	BC035173		2.814672	0.037190267
88	UBE2L3	NM_003347	Hs.108104	2.799959	0.007153104
89	FLJ44342	XR_109412	Hs.710026	2.795932	0.020932825
90	MAFB	NM_005461	Hs.169487	2.793245	0.021591975
91	RTN4RL1	NM_178568	Hs.22917	2.782866	0.026224098
92	CLU	NM_001831	Hs.436657	2.778493	0.01286338
93	ARSA	NM_000487	Hs.88251	2.759177	0.014112548
94	PABPN1L	NM_001080487	Hs.730522	2.755397	0.023294839
95	PAM	NM_000919	Hs.369430	2.748703	0.021098211
96	TUBE1	NM_016262	Hs.34851	2.74672	0.026279342
97	SEL1L3	NM_015187	Hs.479384	2.730905	0.015757567
98	ITGB1	NM_133376	Hs.643813	2.728432	0.037190267
99	LAMB3	NM_001017402	Hs.497636	2.69229	0.025277627
100	HERPUD1	NM_014685	Hs.146393	2.653862	0.009796372
101	S100P	NM_005980	Hs.2962	2.645026	0.007938025
102	HLA-A	NM_002116	Hs.181244	2.633015	0.025789897
103	NOV	NM_002514	Hs.235935	2.612396	0.026813013
104	ASNS	NM_001673	Hs.489207	2.611858	0.012788696
105	APLP2	NM_001642	Hs.370247	2.60488	0.037190267
106	ITPRIP	NM_033397	Hs.523252	2.593298	0.030027078
107	FBLN1	NM_006486	Hs.24601	2.579867	0.030829136
108	KCNK5	NM_003740	Hs.444448	2.573388	0.031740433
109	GLG1	NM_012201	Hs.109731	2.563073	0.044651366
110	SRGN	NM_002727	Hs.1908	2.561892	0.035535315
111	HEXB	NM_000521	Hs.69293	2.558	0.041463231
112	KAT8	NM_182958	Hs.533803	2.543855	0.041463231
113	MAL2	NM_052886	Hs.201083	2.527444	0.042667854
114	SEMA3B	NM_004636	Hs.82222	2.519687	0.049957546
115	TTC28	NM_001145418	Hs.387856	2.497261	0.044224851
116	ZDHHC11	NM_024786	Hs.729226	2.482022	0.046323391
117	GRN	NM_002087	Hs.514220	2.468144	0.013285974
118	HLA-C	M26429	Hs.654404	2.450859	0.030197607
119	HLA-G	NM_002127	Hs.512152	2.435284	0.037190267
120	EFEMP1	NM_001039348	Hs.76224	2.35022	0.039662262
121	SMCR7L	NM_019008	Hs.148677	2.337306	0.046323391
122	MT2A	NM_005953	Hs.647371	2.272632	0.035947396
123	SNAR-H	NR_024342	Hs.717311	-2.574984	1.10E-05
124	SNORA67	NR_002912		-2.6076	0.031740433
125	MFN2	NM_014874	Hs.376681	-2.708317	0.020934481
126	GMPR2	NM_001002000	Hs.368855	-2.724267	0.033618215
127	LOC100652804	XR_132586		-2.945716	0.00677382
128	TRIM16L	NM_001037330	Hs.164324	-3.22221	0.032774839
129	ATP13A2	AY987009	Hs.128866	-3.416452	0.038808666
130	C17orf63	NM_018182	Hs.564533	-3.559995	0.000362262
131	ANGPT4	NM_015985	Hs.278973	-3.586414	0.043579143
132	XLOC_001620	DB077273	Hs.585967	-3.721788	0.000220169

133	CREBL2	NM_001310	Hs.591156	-3.769477	0.030363657
134	MGC11082	BC094703	Hs.659053	-3.829313	0.045939667
135	WARS2	NM_201263	Hs.523506	-3.873603	0.026224098
136	LDHC	NM_002301	Hs.654377	-4.063619	0.002299761
137	FBLIM1	NM_001024215	Hs.530101	-4.095706	0.031767682
138	ASB9P1	NR_033769	Hs.684129	-4.1932	0.015010664
139	VAX2	NM_012476	Hs.249170	-4.255056	0.012690075
140	LOC100131774	XR_110171	Hs.720521	-4.345664	0.015001255
141	LGALS7	NM_002307	Hs.707031	-4.601724	0.005772676
142	NCAPH2	NM_014551	Hs.730607	-4.641252	0.005612474
143	C5orf20	NM_130848	Hs.152477	-4.694513	0.004068832
144	SNORA44	NR_002976	Hs.730337	-4.8549	0.003383891
145	NLK	NM_016231	Hs.208759	-5.270558	0.001182673
146	PROP1	NM_006261	Hs.158301	-6.075803	0.000267435
147	CBX1	NM_006807	Hs.77254	-6.176301	0.000169192
148	PKD1	NM_000296	Hs.546868	-6.264576	0.000153819
149	MTPN	NM_145808	Hs.602015	-7.074359	3.16E-05

Table S7. Genes differentially changed (≥ 2.0 fold & $p < 0.05$) by knockdown of HP1γ in HeLa cells.

No.	Gene Symbol	Genbank	UniGene	HP1γ KD	
				Fold change	p-value
1	TRIM14	NM_033219	Hs.575631	23.067749	0
2	TMCO5A	NM_152453	Hs.179646	19.617327	5.58E-12
3	VWA1	NM_022834	Hs.449009	12.101663	1.80E-08
4	LOC100507055	NM_001195520	Hs.282811	11.177078	8.03E-08
5	MGP	NM_001190839	Hs.365706	8.831503	1.52E-06
6	IL20RB	NM_144717	Hs.61232	8.540477	1.72E-07
7	GDF15	NM_004864	Hs.616962	6.679849	3.49E-11
8	ULBP2	NM_025217	Hs.656778	6.39791	6.82E-05
9	SYT12	AK024280		6.137083	0.000208485
10	TXNIP	NM_006472	Hs.533977	6.00337	3.00E-09
11	ARHGDIB	NM_001175	Hs.504877	5.94771	0.00046033
12	INTS1	NM_001080453	Hs.532188	5.943878	0.000517137
13	SEPP1	NM_005410	Hs.730647	5.600258	0.001156849
14	MMP12	NM_002426	Hs.1695	5.17594	0.003352079
15	PRSS8	NM_002773	Hs.75799	5.14649	0.000513219
16	TP53I13	NM_138349	Hs.514050	5.091151	1.74E-08
17	ESPNL	NM_194312	Hs.127724	5.002369	8.34E-07
18	LOC100506548	NR_037665		4.952158	2.07E-07
19	GSDMB	NM_001165958	Hs.306777	4.929644	0.005588972
20	XLOC_007888	BI754780	Hs.583991	4.818518	0.000645318
21	KIAA1161	NM_020702	Hs.522083	4.776869	0.00773105
22	ZNF862	NM_001099220	Hs.301277	4.653032	0.010122742
23	ZMYND10	NM_015896	Hs.526735	4.601787	0.010849519
24	S100A14	NM_020672	Hs.288998	4.48241	0.005918298
25	TNFRSF10D	NM_003840	Hs.213467	4.381047	0.004061702
26	FBXO32	NM_058229	Hs.403933	4.293741	0.01224485
27	SCARA5	NM_173833	Hs.591833	4.273402	0.004473178
28	LAMB2P1	NR_004405		4.177712	0.027023987
29	PARVB	AK309987	Hs.475074	4.163181	0.002280845
30	SAT1	NM_002970	Hs.28491	4.132636	0.000393201
31	WLS	AB097018	Hs.647659	4.036149	0.013876829
32	CPLX1	NM_006651	Hs.478930	4.019926	0.027526301
33	PDK4	NM_002612	Hs.8364	4.002681	5.98E-05
34	NEAT1	NR_028272	Hs.523789	3.975325	1.00E-05
35	RORC	NM_005060	Hs.256022	3.951889	0.033943719
36	LOC642776	BC003645		3.892199	0.012182389
37	C20orf196	NM_152504	Hs.529340	3.849143	2.29E-05
38	XLOC_009487	BC039105	Hs.639242	3.848241	0.009148399
39	LOC100128071	NM_001167676	Hs.512763	3.797354	0.016325208
40	CLU	NM_001831	Hs.436657	3.716777	0.000164501
41	TSC22D3	NM_004089	Hs.522074	3.704437	5.81E-05
42	ANG	NM_001145	Hs.593708	3.695225	0.035930246
43	XLOC_009723	XR_110375	Hs.729166	3.676487	0.000303272
44	DPYSL3	NM_001387	Hs.519659	3.666461	3.35E-05
45	DKK1	NM_012242	Hs.40499	3.573149	0.013782625
46	LOC100507316	XR_133508		3.474131	0.039662382
47	LOC283454	AK094730	Hs.26605	3.420814	0.026643608
48	PABPN1L	NM_001080487	Hs.730522	3.283721	0.016246269
49	IFITM1	NM_003641	Hs.458414	3.242967	0.046966587
50	CDKN1C	NM_000076	Hs.106070	3.227841	0.000805193
51	LOC100507303	XR_109242	Hs.369728	3.170826	0.000805193
52	TNS4	NM_032865	Hs.438292	2.944427	0.043844615
53	PIM1	NM_002648	Hs.81170	2.912844	0.032519096
54	STK32C	XR_109030	Hs.665362	2.903932	0.028744583
55	C6orf48	NM_001040438	Hs.640836	2.836335	0.004771247
56	XLOC_011102	BX090735	Hs.651496	2.793032	0.00919802
57	LAMB3	NM_001017402	Hs.497636	2.767426	0.013782625
58	EIF4A2	NM_001967	Hs.518475	2.731979	0.010898529
59	ERRFI1	NM_018948	Hs.605445	2.723369	0.03541623
60	UBE2L3	NM_003347	Hs.108104	2.662534	0.010347417
61	LOC644277	XR_110541	Hs.577348	2.620726	0.035930246
62	F11R	NM_016946	Hs.517293	2.575828	0.027526301
63	CKMT1A	NM_001015001	Hs.425633	2.565476	0.027799642

64	STOM	NM_198194	Hs.253903	2.51642	0.043844615
65	ZNFX1-AS1	NR_003605	Hs.356766	2.490531	0.031509883
66	APLP2	NM_001642	Hs.370247	2.470039	0.048440132
67	HSP90AA1	NM_001017963	Hs.525600	-2.109371	0.048946841
68	SNAR-B2	NR_024230	Hs.723095	-2.853741	2.37E-06
69	EIF1AX	NM_001412	Hs.522590	-2.883704	0.002549345
70	HIST2H3A	NM_001005464	Hs.706618	-2.93047	0.002280845
71	SNAR-D	NR_024243	Hs.717309	-2.931731	1.23E-06
72	SNAR-A3	NR_024214	Hs.723094	-2.944123	4.20E-07
73	C5orf20	NM_130848	Hs.152477	-3.251143	0.049718537
74	ZSCAN5A	NM_024303	Hs.177688	-3.346894	0.0483804
75	OBFC2A	NM_001031716	Hs.591610	-3.391887	0.03468658
76	ATF5	NM_012068	Hs.9754	-3.420028	0.035930246
77	SPEG	NM_001173476	Hs.21639	-3.467505	0.048311245
78	SNAR-G2	NR_024244	Hs.717308	-3.479321	1.74E-08
79	FBLIM1	NM_001024215	Hs.530101	-3.693897	0.039662382
80	SNAR-F	NR_004384	Hs.707968	-3.726494	2.65E-09
81	SNAR-H	NR_024342	Hs.717311	-3.746221	6.03E-10
82	GNRH2	NM_178332	Hs.129715	-3.786035	0.01107496
83	CLYBL	AK095506		-3.898999	0.010347417
84	MGC11082	BC094703	Hs.659053	-4.012367	0.014636522
85	VGLL3	NM_016206	Hs.435013	-4.061235	0.012130909
86	LOC100131774	XR_110171	Hs.720521	-4.745354	0.001879623
87	CAST	NM_001042440	Hs.436186	-5.095645	0.000384265
88	HSPA4	NM_002154	Hs.90093	-5.56728	0.000116395
89	SNAR-G1	NR_004383	Hs.621635	-5.9099	1.58E-14
90	CBX3P2	NR_033754	Hs.712861	-7.494164	1.23E-06
91	CBX3	NM_007276	Hs.381189	-10.241708	2.50E-09
